# Evidence for a link between the Atlantic Multidecadal Oscillation and annual asthma mortality rates in the US

**DOI:** 10.1038/s41598-019-48178-1

**Published:** 2019-08-12

**Authors:** Sergio Bonomo, Giuliana Ferrante, Elisa Palazzi, Nicola Pelosi, Fabrizio Lirer, Giovanni Viegi, Stefania La Grutta

**Affiliations:** 1 Istituto per la Ricerca e l’Innovazione Biomedica, National Research Council (CNR-IRIB), Via Ugo La Malfa 153, 90146 Palermo, Italy; 2 Institute for Marine Sciences, National Research Council (CNR-ISMAR), Calata Porta di Massa, 80133 Napoli, Italy; 3 National Institute of Geophysics and Volcanology (INGV), Via della Faggiola 32, 52126 Pisa, Italy; 4 Dipartimento di Scienze per la Promozione della Salute, Materno-Infantile, di Medicina Interna e Specialistica di Eccellenza “G. D’Alessandro”, University of Palermo, Palermo, Italy; 5 Institute of Atmospheric Sciences and Climate, National Research Council (CNR-ISAC), Corso Fiume 4, I-10133 Torino, Italy

**Keywords:** Risk factors, Climate sciences

## Abstract

An association between climatic conditions and asthma mortality has been widely assumed. However, it is unclear whether climatic variations have a fingerprint on asthma dynamics over long time intervals. The aim of this study is to detect a possible correlation between climatic indices, namely the Atlantic Multidecadal Oscillation and Pacific Decadal Oscillation, and asthma mortality rates over the period from 1950 to 2015 in the contiguous US. To this aim, an analysis of non-stationary and non-linear signals was performed on time series of US annual asthma mortality rates, AMO and PDO indices to search for characteristic periodicities. Results revealed that asthma death rates evaluated for four different age groups (5–14 yr; 15–24 yr; 25–34 yr; 35–44 yr) share the same pattern of fluctuation throughout the 1950–2015 time interval, but different trends, i.e. a positive (negative) trend for the two youngest (oldest) categories. Annual asthma death rates turned out to be correlated with the dynamics of the AMO, and also modulated by the PDO, sharing the same averaged ∼44 year-periodicity. The results of the current study suggest that, since climate patterns have proved to influence asthma mortality rates, they could be advisable in future studies aimed at elucidating the complex relationships between climate and asthma mortality.

## Introduction

Asthma is the most prevalent chronic respiratory disease worldwide, affecting more than 330 million people of all ethnic groups throughout all ages^1,2^. Notably, an increasing trend of asthma prevalence is reported in the general population^3^ as well as in children^4^. In the US, in particular, asthma is one of the most common and costly diseases, which about 20 million persons are affected by^5^; furthermore, it is responsible for more than 5000 deaths annually^6^. Adults are four times more likely to die from asthma than children^7^, but childhood asthma death rates have increased by 3.4% per year from 1980 to 1998^8^.

Asthma is thought to be caused by a combination of genetic and environmental factors^9^. The latter can contribute to develop and/or exacerbate the disease and are in great part associated with low air quality conditions, both indoor (e.g. presence of allergens) and outdoor (e.g. allergens and air pollution^10^, high tropospheric ozone levels, and others). Under specific climatic conditions, such as droughts accompanied by dusty conditions and wildfires producing smoke and dust, asthma can get worse. A recent study in the western US, for example, showed that the respiratory-related mortality risk significantly increased by 1.55% (0.17% to 2.95%) during worsening drought conditions^11^ in the period 2000–2013. Other short-term studies focusing on the analysis of temperature variations led to a wide consensus on extremely high temperatures as a risk factor for respiratory-related mortality rates in warmer regions^12,13^. However, assessments on the relationship between long-term changes in the persistence and intensity of temperature- and precipitation-related extremes and asthma death rates are scarce^14^ and scant evidence exists on the impact of temperature and precipitation variations on asthma mortality burden on climatological time scales^15^.

It is worth remembering that climate variations, both in the mean state and in the occurrence of extremes, may result from external factors able to change the Earth’s energy balance but also from internal processes and interactions within or between components of the climate system. External factors can be natural, e.g. variations in the sun, changes in the orbit of the Earth around the sun, volcanic eruptions or anthropogenic, e.g., human related changes in the atmospheric greenhouse gas concentrations or in land use. Increased concentrations of anthropogenic CO_2_ in the atmosphere, for example, have warmed up the planet by about 1 °C since the pre-industrial period, affecting all climate system’s components. The global hydrological cycle, in particular, is getting intensified leading to more severe and prolonged heat waves and droughts, especially in summer, interspersed with periods of intense precipitation and flooding^16^.

Internally induced natural climate variability and change, on the other hand, arise from processes within the atmosphere and ocean particularly, and from interactions between these components. These interactions occur in specific geographical areas but are able to establish “teleconnections” with other regions even over very long distances and affect in this way temperature, rainfall regimes and climate worldwide. Some outstanding examples are represented by teleconnections like El Niño-Southern Oscillation (ENSO), the North Atlantic Oscillation (NAO), the Atlantic Multidecadal Oscillation (AMO), the Pacific Decadal Oscillation (PDO) and others. All of them are expressed as an index whose intensity and sign represent the periodical cycles or phases which characterize the long-term fluctuations of these internal climate variability modes.

As an example, the AMO is a multidecadal fluctuation of sea surface temperatures in the North Atlantic, which has been linked to rainfall and river flow anomalies over the United States^17^, increased drought occurrence over the Southwest and the North-central United States and fewer drought events over Florida during its warm phase^14^. Sometimes different teleconnections interact with each other: the AMO, for example, can also indirectly affect precipitation regimes through the modulation of the influence of ENSO on drought^18^.

While some published studies have assessed the existence of a link between climatic fluctuations, for example in terms of changes in sea surface temperatures or sea level pressure, and the respiratory system^12,15,19^, a few studies exist linking the variations of the indices quantifying internal modes of climate variability and the time variations of respiratory diseases and of asthma specifically. One paper, for example, analysed the association between winter asthma mortality in the UK and the Scandinavia teleconnection pattern (SCA) which has been shown to influence climatic conditions such as precipitation, temperatures and cyclone activity^20^ in Northern Europe during winter.

Starting from the hypothesis that annual asthma death rates could be in part influenced by the occurrence of dry and wet periods, this study aims at investigate a possible link between climate variations in sea surface temperatures expressed by the AMO and PDO teleconnection indices and annual asthma death rates in the US. This study is performed over a relatively long time period (66 years), taking advantage of the availability of US annual asthma death rate data encompassing the years 1950–2015, thus giving us the opportunity to explore, for the first time, the relationship between AMO/PDO and annual asthma death rates over climatological timescales and contributing to fill in one gap in the current state of knowledge on this subject.

### The atlantic multidecadal oscillation and pacific decadal oscillation

The AMO and the PDO are internal modes of climate variability. The AMO is a multi-decadal low-frequency alternation of high and low sea surface temperatures (SSTs) in the North Atlantic^21,22^ with a reported oscillation period of about 65–70 years^21^. Enfield *et al*.^18^ documented a 65–80 year cycle from 1856 to 1999 in North Atlantic SST data. In 2011, Knudsen *et al*.^23^ explored the past 8000 years through proxy records and found a quasi-persistent ∼55- to 70-year AMO signal linked to the ocean-atmosphere internal variability.

The AMO is known to influence rainfall, temperature and pressure in many regions of the Northern Hemisphere^24^, frequency of Atlantic hurricanes^25^, North American climate^26^ and river flows^18^ as well as the hydro-climatic conditions in other areas, like rainfall variability in Northeast Brazil^27^ and occurrence of droughts in the Sahel^28,29^.

The PDO is a pattern of ocean-atmosphere climate variability in the Pacific which appears as warm or cool surface waters in the Pacific ocean poleward of 20°N^30^. Over the past century, the amplitude of this climate pattern has varied irregularly at interannual (time periods of a few years) to interdecadal (time periods of multiple decades) time scales. This climate pattern affects the Northern Hemisphere climate, the North Pacific ecosystem, North American precipitation, stream flow, surface temperature anomalies^31,32^, fluctuations of the Asian monsoon^33^, and a modulation of ENSO^34^. Minobe^35–37^ found that PDO fluctuations were most energetic at periodicities in the 15–25 and 50–70 years bands; Chao *et al*.^38^ found evidence for oscillatory variations at 15–20 and near to 70 years. Willmott *et al*.^39^ jointly analysed the November–April PDO index and surface temperature and precipitation gridded data and found that the warm phases of the PDO tend to coincide with anomalously warm temperatures in northwestern North America, northern South America and northwestern Australia, and anomalously cold temperatures in Eastern China, Korea, Japan, Kamchatka, and the Southeast US and Mexico. Gedalof and Smith^40^ identified 11 regime shifts in the PDO record since 1650, with the most recent occurring in 1976/77. With a 23 years average duration of a regime, they suggested that another shift is expected by around the end of this century. Many of the climate anomalies associated with the PDO are broadly similar to those associated with ENSO phases, though generally not as extreme^30,37,41^.

One recent study^42^ analysed the observed temperature time-series since the beginning of the 20^th^ century in nine climate regions of the US and found that their time variations and oscillations were accurately reproduced by a combination of AMO and PDO oscillations with a monotonic signal associated with anthropogenic CO_2_ warming. The small temperature decrease observed in the period 1938–1974 and the large temperature increase in the period 1980–2000 were thought to be caused, respectively, by the superposition of a downward trend of the oscillatory mode on an upward trend of the monotonic mode and by the superposition of an upward trend of the AMO oscillatory mode on the upward trend of the monotonic mode. Yao *et al*.^43^ found periods of warming slowdown from 1880 to 2012, caused by the superposition of the AMO and PDO oscillations on the steady monotonic warming signal, presumably caused by increasing amounts of atmospheric CO_2_.

Subsequent studies have been focused on the idea that droughts occurred over the contiguous US (in 1996 and in 1999–2002) were associated with North Atlantic warming (positive phase of the AMO) and northeastern and tropical Pacific cooling (negative phase of the PDO). The multidecadal oscillations in the behavior of the North Atlantic Ocean thus plays a crucial role for long-term explanation and predictability of drought frequency in the US^44^.

## Results

### Asthma mortality and climatic data

**Figure 1:**
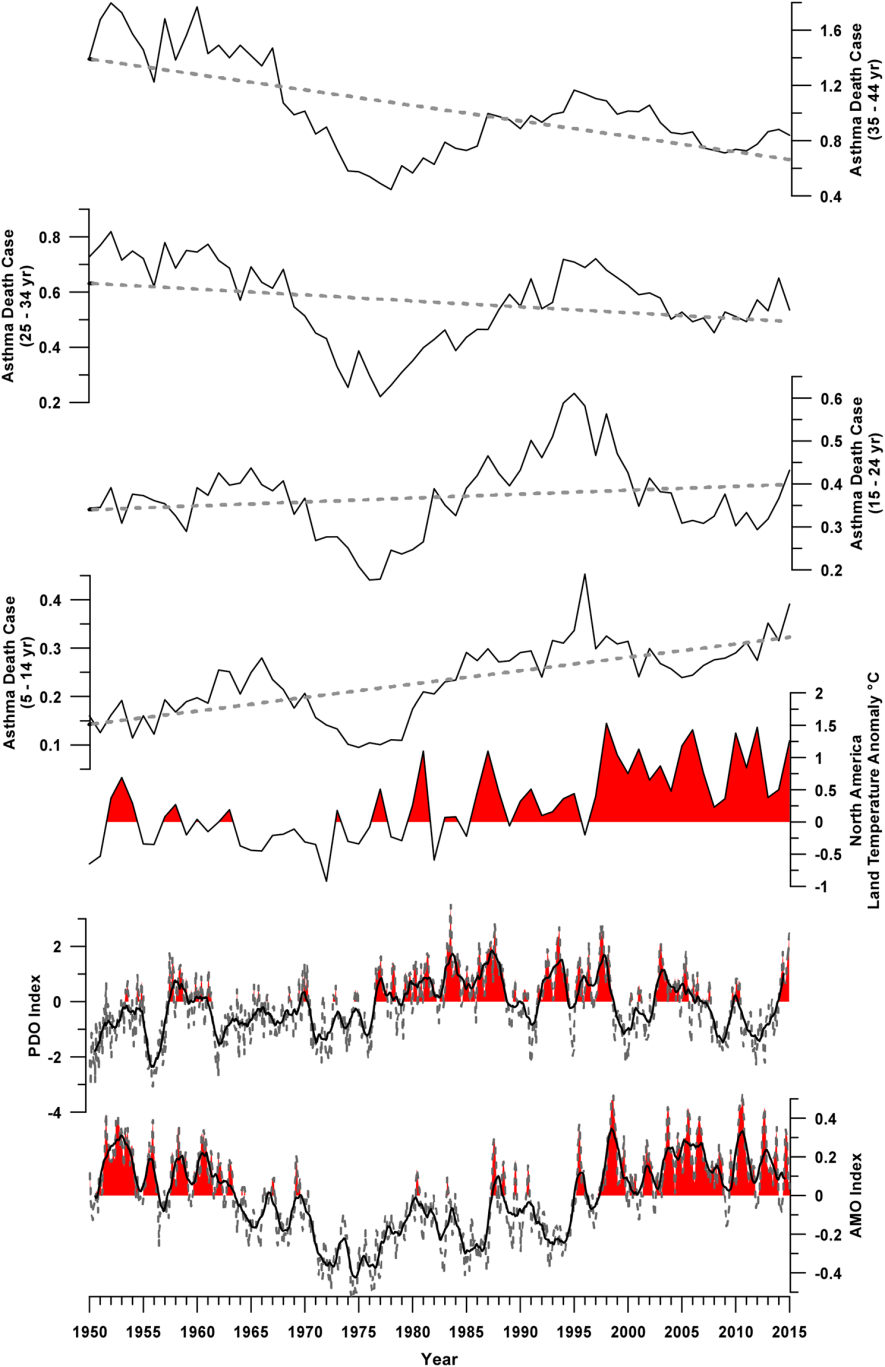
Comparison in time domain between the month (grey dashed lines) and annual mean data (black plain lines) of the Atlantic Multidecadal Oscillation, and Pacific Decadal Oscillation were reported. Annual mean data of the North America Land Temperature Anomaly was reported. Red infill indicate values above zero. Annual asthma mortality death rates for the 4 selected age groups (5–14 yr, 15–24 yr, 25–34 yr, 35–44 yr), grey dashed lines represent linear trends.

North America annual asthma death rates from 1950 to 2015 are shown in the first four plots of Fig. 1, divided by age groups (from the oldest to the youngest, from top to bottom). Mean death rate value over the entire time interval increases from the youngest to the oldest age group (5–14 yr = 0.232; 15–24 yr = 0.369; 25–34 yr = 0.562; 35–44 yr = 1.027). For all age groups, the time-series of annual death rates show a similar oscillatory behaviour but a different trend in the 1950–2015 time period. In particular, the trend is positive for the two youngest categories and negative for the two oldest ones. Less intense positive (negative) trend is observed for the intermediate 15–24 (25–34) years old categories of individuals.

Figure 1 also shows the time-series of North America Land Temperature Anomaly (TA), PDO index and AMO index from 1950 to 2015. The AMO index shows positive values from 1950 to 1964, overall negative values up to 1995, interspersed with short positive events, and again positive values from 1996 to 2015. The PDO index is characterized by negative values from 1950 to 1977 with sporadic positive events, positive values from 1976 to 1999 except in the period 1988–1992, followed by predominantly negative values from 1999 to 2015. The TA time-series exhibits an overall positive trend and an amplification since the 1980s can be observed.

### Correlation matrix between asthma death rates, AMO, PDO, and TA

Table 1 shows the correlation coefficients (and their statistical significance) between asthma death rates for the four age groups, the AMO and PDO indices, and TA. To properly calculate correlations^45,46^, all raw data were first standardized, by subtracting the mean and dividing by the standard deviation, and detrended, by subtracting the linear trend from the original signal. The AMO index is positively and significantly (*p* < 0.05) correlated with asthma death rates for only the 25–34 and 35–44 age groups (25–34 yr, *p* = 2.06 × 10^−8^ r = 0.62; 35-44 yr, *p* = 1.17 × 10^−10^ r = 0.69), as is TA (25–34 yr, *p* = 0.035 r = 0.26; 35–44 yr, *p* = 0.006 r = 0.33). For both AMO and TA, the correlations with the other age groups turn out not be statistically-significant. The AMO index is positively and significantly correlated with TA. The PDO index is positively correlated with the AMO, TA, and the asthma death rates for the two youngest age groups, while negatively correlated with the two oldest age groups; however, none of the PDO correlations turn out to be statistically significant^47^. The four age groups are all significantly (*p* < 0.05) correlated with each other.

**Table 1:** Correlation matrix between Atlantic Multidecadal Oscillation (AMO), Pacific Decadal Oscillation (PDO), North America land temperature anomaly (TA), and asthma death rates for the four age groups.

	AMO	PDO	TA	5/14	15/24	25/34	35/44
AMO		0.342	**5**.**40E**-**08**	0.051	0.235	**2**.**06E**-**08**	**1**.**17E**-**10**
PDO	−0.119		0.308	0.210	0.170	0.398	0.653
TA	*0.610*	0.127		0.663	0.839	**0**.**03515**	**0**.**0065**
5/14	0.241	0.156	0.055		**2**.**88E**-**16**	**3**.**15E**-**10**	**7**.**49E**-**10**
15/24	0.148	0.171	0.025	*0.807*		**3**.**76E**-**10**	**9**.**40E**-**08**
25/34	*0.625*	−0.106	*0.260*	*0.681*	*0.679*		**1**.**77E**-**27**
35/44	*0.693*	−0.056	*0.332*	*0.670*	*0.601*	*0.918*	

Bold and italic numbers indicates the significant *p* values < 0.05 and the relative correlation coefficients respectively.

### Signal analysis results

The CEEMD analysis revealed five intrinsic mode functions (IMFs) plus the trends (IMFs 6) (see Supplementary Information) for all analyzed signals shown in Fig. 1. A first visual analysis of all IMFs shows that from IMF2 to IMF4 the asthma death rate signals (all age classes), AMO, and PDO indices, recorded significative periodicities. Instead, all IMFs1 recorded predominantly noise, and all IMF5 of asthma death rate (all age classes) and TA contain an incomplete cycle. For these reasons we exclude IMFs 1 and 5 from successive analysis.

To obtain analytical results of periodicities recorded in the analyzed signals, we apply REDFIT and weighted wavelet Z-transform (WWZ) on a total of 23 IMFs (see Supplementary Information). The spectra results with periodicities above 95% Confidence Interval (CI) are reported in Table 2.

**Table 2:** Periodicity above 95% CI of asthma death rates for the four age groups, North America land temperature anomaly (TA), Atlantic Multidecadal Oscillation (AMO), and Pacific Decadal Oscillation (PDO), extracted from the IMF2, IMF3, IMF4, and IMF5.

	IMF2	IMF3	IMF4	IMF5
5/14	7.3 (yr) (above 95% CI from 1980 to 2015)	26.4 (yr)	36.3 (yr)	
15/24	7–8 (yr); 13.9 (yr) (above 95% CI from 1965 to 2015)	29.3 (yr)	36.3 (yr)	
25/34	7.1 (yr) (above 95% CI from 1950 to 1990)	29.3 (yr)	36.3 (yr)	
35/44	7.3 (yr) (above 95% CI from 1950 to 1998)	29.3 (yr)	36.3 (yr)	
TA	6.1 (yr) (above 95% CIfrom 1964 to 2015)	11.5 (yr) (above 95% CI from 1985 to 2015) 20.3 (yr) (above 95% CI from 1950 to 1980)	24 (yr) 66 (yr)	
AMO	7.4–8.9 (yr)	17.2–23.2 (yr)	57.0 (yr)	92.8 (yr)
PDO	8.9 (yr) (above 95% CI from 1970 to 2005)	19,0.3 (yr)	46.4 (yr)	66.3 (yr)

**Figure 2:**
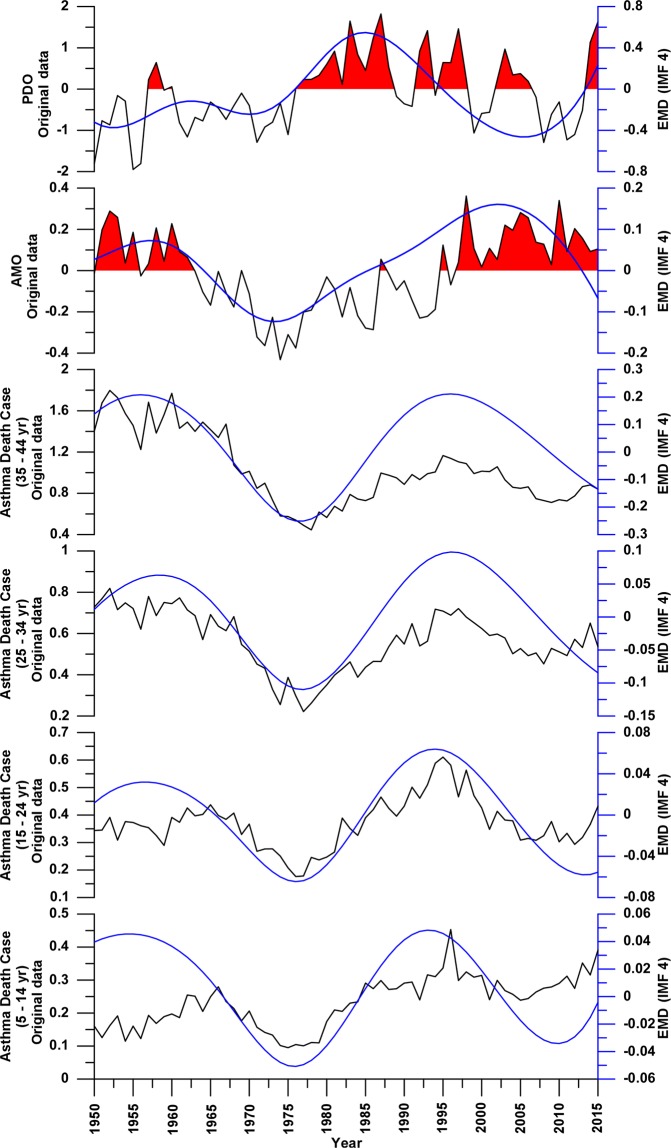
Comparison in time domain between the AMO, PDO, and annual asthma mortality rates raw (black lines) and respectively IMF4 signals (blue lines). Red infill indicate the AMO and PDO values above zero.

**Figure 3:**
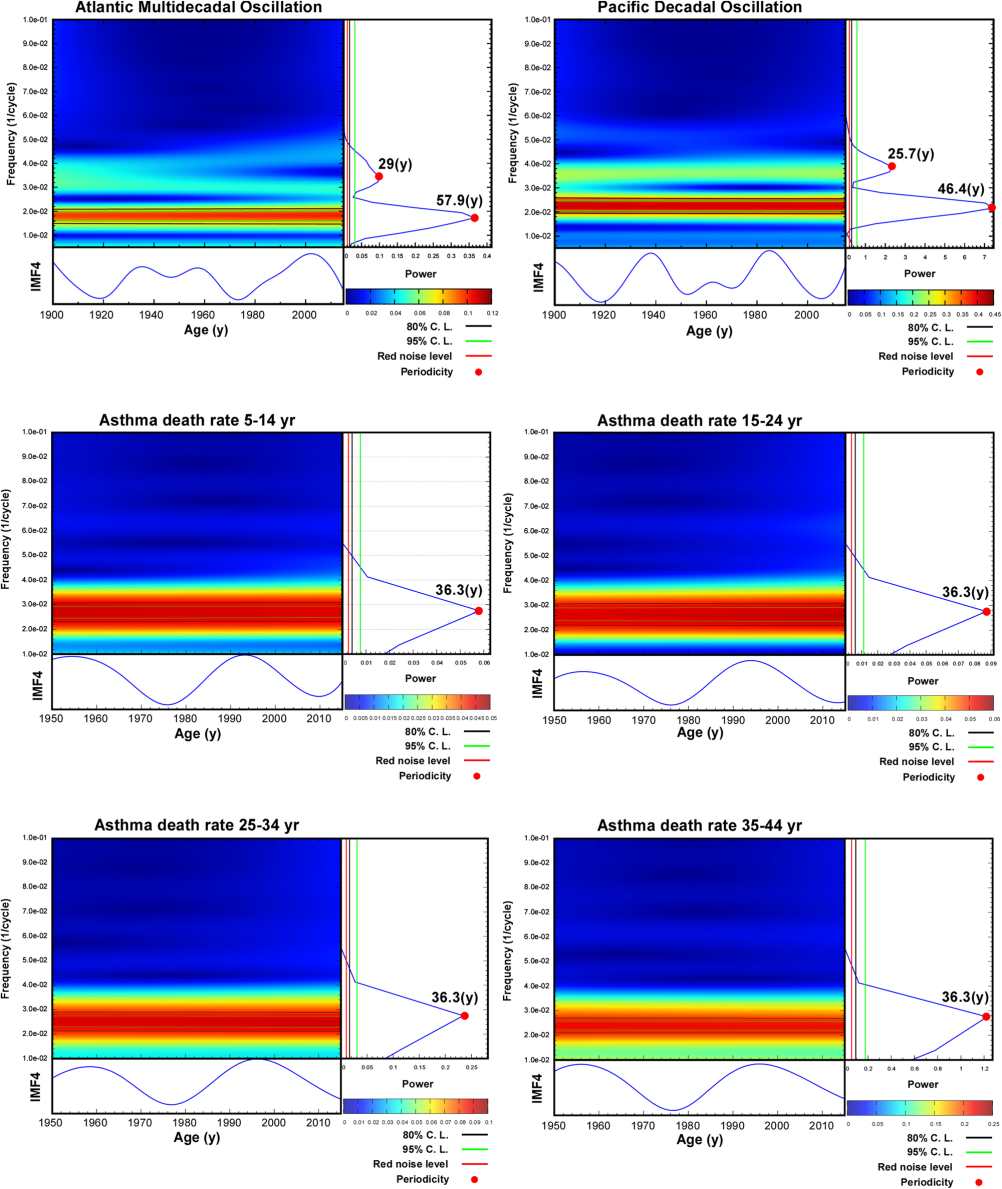
Signal analysis of the AMO, PDO, and the four annual asthma death rate age groups. In the 6 box IMF4 (horizontal boxes, blue line), Lomb-Scargle periodogram (vertical boxes, blue line), and weighted wavelet Z-transform power spectrum were reported. The green and black line represent the 95% and 80% Confident Level respectively. Significantly periodicity (red dot) and relative values expressed in years were reported.

These results show us that only the IMF4 component of all signal (Fig. 2), except for the TA, have a main peaks in the ~37/~57 years periods range, all above the 95% CI and continuous throughout the investigated time window (Fig. 3).

**Figure 4:**
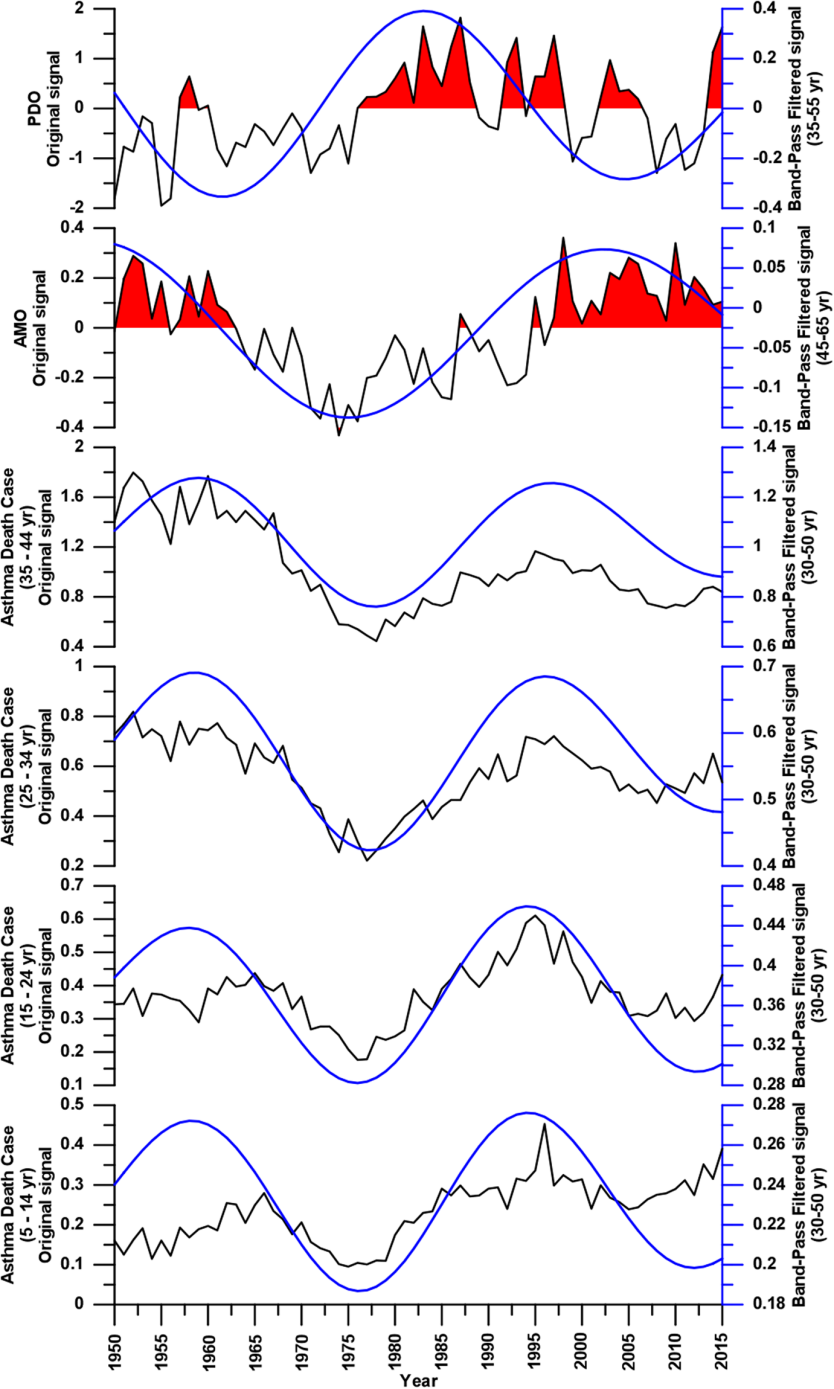
Comparison in time domain between the AMO, PDO, and annual asthma mortality rates raw (black lines) and band-pass signals (blue lines) filtered with the wavelet decomposition and reconstruction method, in a narrow range centered on long-term trend climatic cycles.

Finally, applying a bad-pass filter to the AMO (filter 45–65 years), PDO (filter 35–55 years), and the asthma mortality raw data (filter 30–50 years), all insignificant frequencies were removed in order to obtain a more easy inspection of the relation between the asthma death rates and climatic indices. In Fig. 4, the raw and band-pass filtered data of all age groups death rates, AMO, and PDO are depicted. The visual inspection of all significant data suggests synchronicity between AMO and asthma death rates for all age groups, highlighting a slight desynchronisation effect starting from ∼1980. With regard to PDO *vs* AMO and asthma death rates, negative synchronicity, centered at ∼1960 and ∼2000 respectively, occurs. During the 1980–85 time interval, the high positive PDO period seems to show null relationship with other data.

## Discussion

This is the first study comparing historical US annual asthma death rates for four age groups, from childhood (5–14 yr) to adulthood (35–44 yr), with relevant climatic indices, which are known to modulate drought periods in this geographic area. Droughts conditions are among the main contributors to environmental factors leading to or exacerbating asthma.

The studied period covers a long time-span (66 years), from 1950 to 2015, during which an increasing trend in surface air temperature has been observed and the historical records of precipitation, streamflow and drought indices have pointed toward increased aridity since 1950 over many land areas^48^, including the regions considered in this paper.

Mean asthma death rates in the US have revealed a gradual increase, from the younger age-groups to the older ages^49,50^; this might be due to an increase in comorbid conditions (e.g. obesity) aggravating the asthma management or possibly causing death events^51^. Moreover, all age groups share the same increase/decrease sequences in asthma death rates throughout the 1950–2015 time period but a different long-term trend^52^. Since a long time-span of asthma mortality rate data in US has been encompassed for the first time, our findings cannot be adequately compared to previous results. Nonetheless, results obtained for shorter time intervals (e.g., 1968–1987^52^) are in agreement with our findings on increasing/decreasing sequences and trends.

The observed variations in asthma death rates cannot be an effect of the four revisions of the International Classification of Disease codes (ICD) occurred in the last 56 years (see Methods), since they do not coincide with sharp inversion or trend modification which has occurred. Instead, a combination of different factors including increasing prevalence of asthma, changing patterns of both disease severity and medical care, as well as an enhanced recognition of the disease might be the major drivers of the observed sequence of fluctuations^52^. In particular, when considering the introduction of bronchodilators from 1960’s and the inhaled steroid treatment from 1990’s, no evident effects on US annual asthma death rates could be recognized. Noteworthy, an increase trend of annual asthma mortality can be observed starting from 1980’s and encompassing the 1990’s up to 1996 in all the age groups. According to previous studies, this finding might be attributed to different factors relative to prescription patterns^53^, medication misuse, underuse, overuse, as well as toxic effects^54^.

On the other side, environmental variables may play a role that could mainly be observed when long time intervals are taken into account.

Our results pointing toward a correlation between increase/decrease sequences of asthma death rates and the AMO index oscillation may indicate a phenomenon previously unrecognized in the link between climate variability and respiratory events. Indeed, a comparable evolution of the AMO index and the fluctuations of annual asthma mortality rates for the four age groups has been observed in the 30-year period 1950–1980. Periods of maximum (minimum) in asthma death rates correspond to periods of positive (negative) values in the AMO index, thus suggesting the AMO, through its links to drought events, as a possible risk factor for asthma mortality in the contiguous US. Additionally, it is known that the 1945–1957 drought period, a European-to-North America continent event recorded by several different datasets, indices, and proxies (e.g.: Global Historical Climatic Network observation-based dataset, Palmer Drought Severity Index, tree rings^55–57^; Reworked Coccoliths^58^ and references therein), was for the first time correlated to US asthma death rates. After 1980, a combined superimposing effect of increasing TA and positive PDO occurred. This effect impairs the visual correlation between mortality data and AMO index for the last 35 years. In order to bypass this problem and to highlight the common cyclicity, the EMD analysis allowed us to find main peaks centred at ~57 and ~46 years (mean ∼51 yr) for AMO and PDO, respectively. This result is in agreement with previous literature data^23,42^. In the mortality IMF4 signal, main peaks centred at ~37 years were found from the younger to the older age groups. The comparison of all IMF4 data in respectively long-term frequency band shows that the asthma death rates signals are in phase with the AMO oscillations during the 1950–1980 time interval, where maxima in mortality correspond to positive values in AMO signal. After 1980, a slight desynchronization of about 5–7 years has been recognized, probably related to the co-occurrence of a long positive phase of the PDO index, the 1980/90 abrupt climatic shift, and a period characterized by the two largest volcanic eruption of the century, El Chichón and Pinatubo, occurred in 1983 and in 1991, respectively.

The global climate shift in the late 1980s was observed in the atmosphere^59,60^, ecosystems^61^ and human-social systems^62^. Three regime shifts (1970s, 1980s and 1990s), distinguished by marked increases in temperatures or by abrupt temporal changes across different biophysical systems, have been identified in the last few decades^63–68^. Whilst documented at ocean basin or regional scales, the mechanisms behind these events, their environmental interactions, and the synchrony and scale of their effects around the globe are poorly understood. Thus, there is a considerable research gap with many disparate observations by different scientific disciplines, without a comprehensive overall assessment. In addition to its modulation of the carbon cycle^69^; diseases (*Vibrio cholerae*^70^); biotic, physical and chemical land components^71,72^; freshwater^73^; precipitation^74^; marine^75,76^; cryospheric^77^ and atmospheric^59,60^ systems, herein we hypothesize that the 1980 shift might have modulated also the US asthma mortality cyclicity.

This study certainly has some limitations, such as not having considered gender classifications or having limited the analysis to a very large area without focusing on small regions, potentially useful to highlight the contribution of local climate or other local effects.

On the other hand, the strength of this research lies in the long time interval which is investigated, allowing to better identify periodical signals, and in the application of a novel robust methodology (EMD) for studying asthma mortality rates.

## Conclusions

The interactions among AMO, PDO, and the climatic conditions including the 1980–90 climatic shift, may have influenced and shaped the asthma mortality rate fluctuations in the US during the last 66 years. This result is supported by the finding of a common mean periodicity of about 44 years among the analysed variables, which introduces some new elements in the research on asthma epidemiology, though cyclical fluctuations have been already documented in the epidemiology of other diseases (e.g., pneumoniae^78^; influenza^79^).

Furthermore, since drought variations in US have been shown to be controlled by AMO and PDO indices, they may be hypothesized as an emerging risk factor for asthma mortality. This is in agreement with the recent statement of the Global Asthma Network: “Environmental factors are much more likely than genetic factors to have caused the large increase in the number of people in the world with asthma, but we still do not know all the factors and how they interact with each other and with genes”^80^.

At last, the results of the current study suggest that patterns of climate variability may be emerging risk factor for asthma mortality rates. Thus, it would be advisable to develop future correlative studies including the aforementioned climatic indices in order to elucidate the complex relationships between climatic factors and asthma mortality. Finally, further studies are envisaged to test the applicability of our methodology also in different geographic areas and confirm or extend our findings.

## Methods

### Asthma mortality data

The annual asthma mortality dataset was downloaded from the US Centers for Disease Control and Prevention - National Center for Health Statistics - National Vital Statistics System website (1950 to 1998 data downloaded from https://www.cdc.gov/nchs/nvss/mortality/hist290.htm - 1999 to 2015 data downloaded from https://www.cdc.gov/nchs/data_access/Vitalstatsonline.htm#Mortality_Multiple). This dataset is a compilation of mortality data by 10 yr-age groups, race, gender, and cause of death (according to the International Statistical Classification of Disease and Related Health Problems –ICD code), as reported annually.

Asthma deaths were identified using the following ICD codes: ICD-7 241 (from 1950 to 1967), ICD-8/9 493 (from 1968 to 1998), and ICD-10 J45/46 (from 1999 to 2015). Population age classes were categorized into children (5–14 years), youth (15–24 years), young adults (25–34 years), and middle-aged adults (35–44 years), following the World Health Organization classification. Older age groups were not taken into account in view of the possible lack of precision of death certificates for asthma in such ages. To study long-term trends, the crude asthma mortality rates (n/100000) for the 4 age groups were calculated^49^.

### Climatic indices

The detrended monthly mean AMO and of PDO data from 1900 to 2015 were downloaded from the National Oceanic and Atmospheric Administration (https://www.esrl.noaa.gov/psd/data/timeseries/AMO/ - https://www.esrl.noaa.gov/psd/gcos_wgsp/Timeseries/PDO/). The AMO and PDO annual mean values were calculated by applying a 12 points running average. Annual mean data of the North America land temperature anomalies (TA, 1950–2015) were downloaded from https://www.ncdc.noaa.gov/cag/global/time-series/northAmerica/land/ytd/12/1950–2015.

### Signal analysis

In order to single out characteristic periodicities in the time-series which we analysed, the analysis of the non-stationary (frequency changes with time) and non-linear signals was performed by applying the Empirical Mode Decomposition algorithm (EMD) by Huang *et al*.^81^.

The Ensemble Empirical Mode Decomposition (EEMD) and its complete variant (CEEMD) are adaptive, noise-assisted data analysis methods that improve on the ordinary Empirical Mode Decomposition (EMD) by Huang *et al*.^81^. This decomposition provides a powerful method to look into the different processes behind a given time series data, and provides a way to separate short time-scale events from a general trend.

Empirical mode decomposition is a form of adaptive time series decomposition method where the basis functions are derived from the signal itself, while in some standard forms of spectral analysis methods like Fourier and wavelet analysis, the basis functions are fixed as sine and cosine for the first and as mother wavelet functions for the second. The decomposition process produce IMFs that are singular function representing an oscillatory mode with one instantaneous frequency that needs to satisfy two criteria:

- In the whole time series, the number of extrema and the number of zero crossings must be either equal or differ at most by one;
- At any point in the time series, the mean value of the envelopes which is defined by local maxima (upper envelope) and local minima (lower envelope) is equal to zero.

This decomposition technique rests on the assumption that any complicated signal can be decomposed into a finite and often small number of components called “Intrinsic Mode Functions” (IMF)^81^, each of them representing an embedded characteristic simple oscillation on a separated time-scale. The data were detrended prior to analysis. We have added in the Supplementary Materials a complete discussion of EMD analysis and its upgrades, as well as of REDFIT and Foster’s wavelet spectral technique.

The IMF components are analysed with “REDFIT”, an evolution of the Lomb-Scargle periodogram^82–84^, and Foster’s^85^ weighted wavelet Z-transform.

With the purpose of comparing the dominant periodicities recorded in the asthma death rates with the same order periodicities documented in the reference global signal (AMO and PDO), we applied a band-pass filter using the wavelet multi-level decomposition and reconstruction technique, which is invertible and thus suitable for filtering data. In particular, we used the multiresolution analysis (MRA) algorithm to decompose a signal into scales with different time and frequency resolution organized according to a hierarchical scheme^86^.

## Supplementary information

Evidence for a link between the Atlantic Multidecadal Oscillation and annual asthma mortality rates in the US
