# Supplementary material for: Evidence for a link between the Atlantic Multidecadal Oscillation and annual asthma mortality rates in the US
Source: Sci Rep. 2019 Aug 12;9:11683. doi: 10.1038/s41598-019-48178-1 (PMC6690970; doi:10.1038/s41598-019-48178-1)
Supplement: Supplementary file 1 — Evidence for a link between the Atlantic Multidecadal Oscillation and annual asthma mortality rates in the US [file 41598_2019_48178_MOESM1_ESM.pdf]

Supplementary information for

Evidence for a link between the Atlantic Multidecadal Oscillation and annual asthma mortality rates in the US

Sergio Bonomo[1-2-3], Giuliana Ferrante[4], Elisa Palazzi[5], Nicola Pelosi[2], Fabrizio Lirer[2], Giovanni Viegi[1], Stefania La Grutta[1]

1 Institute of Biomedicine and Molecular Immunology "Alberto Monroy", National Research Council (CNR-IBIM), Via Ugo La Malfa 153, 90146 Palermo, Italy.
2 Institute for Marine Sciences, National Research Council (CNR-ISMAR), Calata Porta di Massa, 80133- Napoli, Italy.
3 National Institute of Geophysics and Volcanology (INGV), Via della Faggiola 32, 52126, Pisa.
4 Dipartimento di Scienze per la Promozione della Salute, Materno-Infantile, di Medicina Interna e Specialistica di Eccellenza "Giuseppe D'Alessandro", University of Palermo, Italy.
5 Institute of Atmospheric Sciences and Climate, National Research Council (CNR-ISAC), Corso Fiume 4, I-10133 Torino, Italy
Corresponding author: giuliana.ferrante@unipa.it

Signal Analysis Methods

In order to extract periodicities recorded in a non-linear and non-stationary (frequencies change over time) time-series, we need a data analysis technique with the ability to decompose they into a finite amount of AM-FM components, called Intrinsic Mode Functions (IMFs), in the time domain itself.
The Ensemble Empirical Mode Decomposition (EEMD) and its complete variant (CEEMD) are adaptive, noise-assisted data analysis methods that improve on the ordinary Empirical Mode Decomposition (EMD) by Huang et al.[1]. This decomposition provides a powerful method to look into the different processes behind a given time series data, and provides a way to separate short time-scale events from a general trend.
Empirical mode decomposition is a form of adaptive time series decomposition method where the basis functions are derived from the signal itself, while in some standard forms of spectral analysis methods like Fourier and wavelet analysis, the basis functions are fixed as sine and cosine for the first and as mother wavelet functions for the second. The decomposition process produce IMFs that are singular function representing an oscillatory mode with one instantaneous frequency that needs to satisfy two criteria:

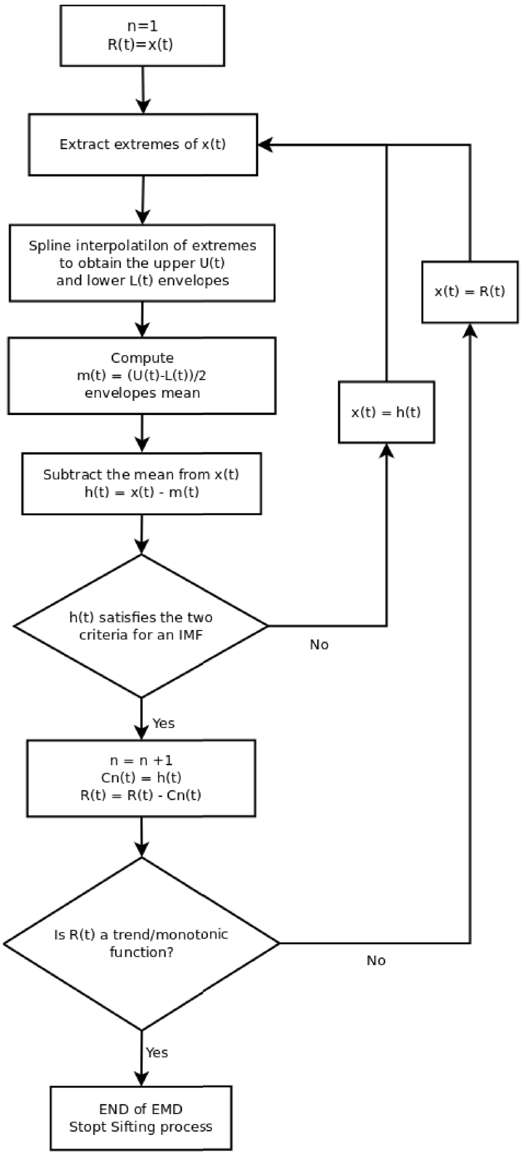

- In the whole time series, the number of extrema and the number of zero crossings must be either equal or differ at most by one;
- At any point in the time series, the mean value of the envelopes which is defined by local maxima (upper envelope) and local minima (lower envelope) is equal to zero.

The EMD theory says that any signal $x(t)$can be decomposed using the following equation[1]: $x(t) = \sum_{i=1}^{n} IMF_i(t) + R_n(t)$ where $IMF_i(t), (i = 1,2, \dots, n)$ are the Intrinsic Mode Functions (IMF) and $R_n(t)$is the residue-trend component.

The computational steps to extracting IMFs via EMD is based on the "Sifting algorithm", which is an iterative method detailed as follows:

Step 1: Identify all the local maxima and minima of the signal $x(t)$.

Step 2: Interpolate the local extrema using cubic spline to obtain the upper $U(t)$ and the lower $L(t)$envelopes.

Step 3: Calculate the local mean value of the upper and the lower envelopes $m(t) = (U(t) + L(t))/2$.

Step 4: Subtract the local mean $m(t)$ from the from the original signal: $h_1(t) = x(t) - m(t)$.

Step 5: Replace the signal $x(t)$ by$h_1(t)$and check if $h_1(t)$satisfies the two criteria for an IMF. If not reiterate Steps 1–4 until the $h_1(t)$ satisfies the IMF criteria.

Assuming that after i times of iteration, the conditions are satisfied then $C_1(t) = h_1(t)$ becomes the first IMF and so on. When $R_n(t) = h_n(t)$, $R_n(t)$ represents a residuum (trend-monotone function), we stop the sifting process; if not, increase n and start at step 1 again to extract another IMF.

The decomposition process actuated with EMD, starts with the sequential extraction of higher frequencies IMF versus lower, until the last residual trend.

The EMD algorithm has some limitations in decomposing signals where amplitude-frequency ranges are too close to each other, these closely spaced components will be intermittent in the signal. This phenomenon, called mode-mixing problem, is amplified when sifting algorithm use all the local extrema to construct the interpolated envelopes, owing to the sensitivity of interpolation methods, stop criterion of IMF, and the end effect. Everything does yes that the intermittent signals cannot be separated from the IMF.

The mode-mixing phenomenon manifests when the oscillations with different time scales are preserved in one IMF, or, from a different view, the oscillations with the same time scale are sifted into different IMFs.

To overcome the mode mixing obstacle, an extension to EMD algorithm was proposed by Wu and Huang[2] and it is called Ensemble EMD (EEMD). It performs EMD over an ensemble of Gaussian white noise assisted data. The EEMD consists of adding different realizations of white noise to the original data $x(t)$in several trials. Since the added noise is different in each trial, the resulting IMFs do not exhibit any correlation with the corresponding IMFs from one trial to another. If the number of trials is sufficiently high, the added noise can be eliminated by ensemble averaging of the obtained IMFs related to the different trials (stacking). EEMD algorithms can be described as:

1. Add series of white Gaussian noise $w_i(t)(i = 1, \dots, L)$ to the original signal $x(t)$ and generate $x_i(t) = x(t) + w_i(t)$;

2. Derive a set of IMFs $C_{ij}(t)(j = 1, \dots, n)$ and residues $R_i(t)(i = 1, \dots, L)$ by decomposing each of $x_i(t)$ applying EMD; where, $C_{ij}(t)$is the jth IMF of the ith trial;

3. Repeat the above steps until i > L;

4. Average over the ensemble to obtain the final IMF $C_j(t)(j = 1, \dots, n)$.

Although EEMD alleviates the effect of mode mixing, noise will remain in the corresponding IMFs if the ensemble number is small. To eliminate the residue of added noise in IMFs, a Complete EEMD (CEEMD) algorithm, developed by Torres et al.[3] was introduced. The noise residue was reduced by adding a fixed percentage of positive and negative white noise to the original data generating two sets of ensemble IMFs. The final IMF components are the means of all corresponding IMF components. The CEEMD algorithm process can be described as follows:

1. Add a pair of opposite phase Gaussian white noises $w_i^{\pm}(t)(i = 1, \dots, L)$ to $x(t)$ with the same amplitude, generating two signals as follows: $\begin{matrix} x_i^+(t) = x(t) + \omega_i^+(t) \\ x_i^-(t) = x(t) + \omega_i^-(t) \end{matrix}$;

2. Repeat Step 1, and decompose each new data $x_i^+(t)$ and $x_i^-(t)$ using the EMD algorithm;

3. Obtain two sets of IMFs $C_{ij}^+(t)$ and $C_{ij}^{\pm}(t)(j = 1, \dots, n)$;

4. Obtain the final IMFs $C_j(t)(j = 1, \dots, n)$ by averaging $C_{ij}^+(t)$ and $C_{ij}^{\pm}(t)(j = 1, \dots, n)$ using this equation $C_j(t) = \frac{1}{2L}\sum_{i=1}^{2L} C_{ij}(t)$; where $C_{ij}(t)$ represents the j-th IMF of the i-th iteration.

All the IMF components extracted from the asthma death signals and the reference global signals are analyzed with "REDFIT" (Schulz & Mudelsse[84]), an evolution of the Lomb-Scargle periodogram[4,5], and Foster's[6] weighted wavelet Z-transform.

"REDFIT" is a specialized tool to analyze unevenly sampled signal $X(t_i)$, where $t_i = t_1, t_2, \dots, t_N$.

The REDFIT modified periodogram, as a function of the frequency ω, is so defined:

$$P_X(\omega) = \frac{1}{2}\{\frac{[\sum x(t)\cos(\omega(t-\tau))]^2}{\sum \cos^2(\omega(t-\tau))} + \frac{[\sum x(t)\sin(\omega(t-\tau))]^2}{\sum \sin^2(\omega(t-\tau))}\};$$

where τ is defined as: $\tan(2\omega\tau) = \frac{\sum\sin(2\omega t)}{\sum\cos(2\omega t)}$.

When $P_X(\omega)$ is defined in this manner, it has several useful properties which the usual discrete Fourier transform does not have. The inclusion of the τ constant, that is a frequency dependent time delay, ensure to make the periodogram insensitive to time shift.

A more import property of modified periodogram $P_X(\omega)$ is defined so that if the time series $X(t_i)$ is purely white noise, then the power in $P_X(\omega)$ follows an exponential probability distribution function. This exponential distribution provides a convenient estimate of the "false alarm probability" that says if a given peak is a true periodicity, or whether it is the result of randomly distributed noise. The periodogram $P_X(\omega)$ has the desired exponential PDF only when it is normalised by the total variance of the data[5], and this result is still valid for signals with an uneven time-spacing.

The "false alarm probability" works good only if the noise contained in the signal is "white", generally, stratigraphic/paleoclimatic signals are characterized by "red-noise" that in the periodogram is showed by continuous decrease of spectral amplitude with increasing frequency[7,8]. Therefore, for red-noised signals the estimate of confidence levels in Lomb-Scargle periodogram, cut off high frequencies spectral components and enhance low frequencies false peaks. A valid solution at this problematic is the utilization of a first-order autoregressive AR(1) process to explain this red-noise signature[9], this solution was implemented by Schulz and Mudelsee[7] in the "REDFIT" software that estimates the AR(1) parameters directly from unevenly spaced signal. The estimated AR(1) model is then transformed from the time domain into the frequency domain. Comparison of the modified periodogram of the our signal with the spectrum of the AR(1) model allows to test the hypothesis that the analyzed signal is consistent with a red-noise AR(1) model. Once it is made this test we can:

- to correct our signal Lombe-Scargle spectrum because it is biased, in particular spectral amplitudes at the high frequency end of the spectrum are often over-estimated;
- to estimate the confidence levels because now the PDF of the spectrum, at each frequency, follows a χ^2 distribution.

The Foster's[4] weighted wavelet Z-transform, that handle irregularly sampled signals, is defined as a suitable weighted projection onto three trial function giving the Weighted Wavelet Z transform (WWZ) and the Weighted Wavelet Amplitudes (WWA).

The concept of "projection" is applied in most time series analysis methods and consists to model the observed data as a linear combination of trial functions and to adjust their coefficients and parameters so as to minimize the sum of the squared errors.

The inner product is the main tool to project the observed data onto any subspace of the function space. In fact, we can project the sampled time series vector onto the subspace of the sampling space, using a set of n trial functions $\varphi_a(t), a = 1,2,3.....,n$ that define a set of trial vectors $|\varphi_a(t)\rangle = [\varphi_a(t_1), \varphi_a(t_2), \varphi_a(t_3),......, \varphi_a(t_N)]$, linearly independent.

Such subspace of trial vectors, was called by Foster "model space" of dimension $n \leq N$.

Each vector $|y\rangle$ in the model space is a linear combination of trial functions with constant coefficients y_a; $|y\rangle = \sum_a y_a|\varphi_a\rangle$. Now, the target of the projection is to compute the coefficients y_a, for which the vector $|y\rangle$ minimizes the sum of the squared residuals. To compute these coefficients, we use the "inner product" of two model space vectors $|y\rangle$ and $|z\rangle$ with coefficients y_a and z_b $\langle y|z\rangle = \sum_a \sum_b y_a z_b \langle\varphi_a|\varphi_b\rangle = \sum_a \sum_b S_{\text{ab}} y_a z_b$, where S_{ab} is the *Super-matrix* $S_{\text{ab}} = \langle\varphi_a|\varphi_b\rangle$, that represents the matrix of inner products of the trial functions. To obtain the best fit y_a coefficients of the trial vectors, we multiply the inverse of the *S-matrix* by the vector of inner products of the trial function with our time series data: $y_a = \sum_b S_{\text{ab}}^{-1}\langle\varphi_b|x\rangle$. Well-know these coefficients, we can finally compute the model function $|y\rangle$ and its variation: $V_y = \langle y|y\rangle - \langle 1|y\rangle^2$.

The WWZ-WWA transform is a typical example of function-space projection, where to treat unevenly sampled data we need a projection onto complex and weighted trial function as the abbreviated Morlet mother wavelet:

$\varphi(t) = e^{i\omega(t-\tau)-c\ \ ^2(t-\tau)^2}$,

that we can split in the trial function part: $\varphi(t) = e^{i\omega(t-\tau)}$,

and in the statistical weights part: $w_a = e^{-c\ \ ^2(t_a-\tau)^2}$.

Now, to compute the weighted projection coefficients y_a, we calculate the inner product between the following three trial functions: $\varphi_1(t) = 1(t)$; $\varphi_2(t) = \cos(\omega(t-\tau))$; $\varphi_3(t) = \sin(\omega(t-\tau))$, and the sampling space vector $|x(t)\rangle$, using a weights w_a.

$y_a = \frac{\sum_{a=1}^{3}\sum_{\beta=1}^{N} S_{\text{ab}}^{-1} w_a \varphi_a(t_\beta) x(t_\beta)}{\sum_{a=1}^{3} w_a}$; where; $S_{\text{ab}}^{-1} = \langle\varphi_a|\varphi_b\rangle^{-1}$, a=1,2,3.

This enables to write the weighted variation (V_y) of the model function as:

$$V_y = \frac{\sum_a w_a y^2(t_a)}{\sum_b w_b} - \left[\frac{\sum_a w_a y(t_a)}{\sum_b w_b}\right]^2 = \langle y|y\rangle - \langle 1|y\rangle^2 .$$

Similarly, we define the weighted variation (V_x) of the sampling space vector $x(t_i)$, corresponding at the time series data, as:

$$V_x = \frac{\sum_a w_a x^2(t_a)}{\sum_b w_b} - \left[\frac{\sum_a w_a x(t_a)}{\sum_b w_b}\right]^2 = \langle x|x\rangle - \langle 1|x\rangle^2.$$

Now we can define the weighted wavelet transform as: $\text{WWT} = \frac{(N_{\text{eff}}-1)V_y}{2V_x}$,

where: $N_{\text{eff}} = \frac{(\sum w_a)^2}{\sum w_a^2} = \frac{[\sum e^{-c\omega^2(t-\tau)^2}]^2}{\sum e^{-2c\omega^2(t-\tau)^2}}$ and represents the effective number of data points contained in each analyzing wavelet function.

The WWT now defined is true only when the time series data is random (white) noise, instead, if our signal holds a frequency, we will have a false peak shifted towards lower frequency. This phenomenon is due to large wavelet window dimension at lower frequency so it samples more data points, therefore if N_{eff} is large the WWT values increase with decreasing ω.

To have a real estimate of the frequency of significant peaks, we need to insert a test statistic less sensitive to the effective number of data. This statistic developed by Foster and called Z-statistic, convert the WWT in WWZ (Weighted Wavelet Z-transform), with this expression: $\mathrm{WWZ}(\omega, \tau) = \frac{(N_{\mathrm{eff}}-3)V_y}{2(V_x-V_y)}$.
Values of the WWZ are approximately an F-statistic with $N_{\mathrm{eff}} - 3$ and 2 degrees of freedom, and expected value 1. This statistic evaluates the normalized quotient of the weighted variation of the projected data and the projected model function related to pure noise. For each couple (ω, τ) the Null hypothesis (signal is pure noise), is tested and if rejected we have the certainty that the frequency ω at the age τ, is a real component of the signal above the background noise.

The WWZ is a powerful tool to identify signal frequency and their position, but it can't measure signal amplitude. To calculate the amplitude of the corresponding periodic fluctuation we define the Weighted Wavelet Amplitude: $\mathrm{WWA} = \sqrt{(y_2)^2 + (y_3)^2}$; where y_2 and y_3 are the expansion coefficients for the sine and cosine functions. Therefore, if the signal is periodic at the frequency being tested, WWA gives the real semi-amplitude of the corresponding best-fit sinusoid.

Wavelet Threshold Denoising Model

With the purpose of comparing the dominant periodicities recorded in the asthma death rates with the same order periodicities documented in the reference global signal (AMO and PDO), we applied a band-pass filter using the wavelet multi-level decomposition and reconstruction technique, which is invertible and thus suitable for filtering data. In particular, we used the multiresolution analysis (MRA) algorithm to decompose a signal into scales with different time and frequency resolution organized according to a hierarchical scheme[10].

References

1. Huang, N. E. *et al.* The empirical mode decomposition and Hilbert spectrum for nonlinear and nonstationary time series analysis. *Proc. Roy. Soc. London* **A454**, 903–995 (1998).
2. WU, Z. & HUANG, N. E. ENSEMBLE EMPIRICAL MODE DECOMPOSITION: A NOISE-ASSISTED DATA ANALYSIS METHOD. *Adv. Adapt. Data Anal.* (2008). doi:10.1142/s1793536909000047
3. Torres, M. E., Colominas, M. A., Schlotthauer, G. & Flandrin, P. A complete ensemble empirical mode decomposition with adaptive noise. in *2011 IEEE International Conference on Acoustics, Speech and Signal Processing (ICASSP)* 4144–4147 (IEEE, 2011). doi:10.1109/ICASSP.2011.5947265
4. Lomb, N. R. Least-square frequency analysis of unequally spaced data. *Astrophys. Sp. Sci.* **29**,

447–462 (1976).

5. Scargle, J. D. Studies in astronomical time series analysis, II Statistical aspects of spectral analysis of unevenly spaced data. *Astrophys. J.* **263**, 835–853 (1982).

6. Foster, G. Wavelets for period analysis of unevenly sampled time series. *Astron. J.* **112**, 1709–1729 (1996).

7. Schulz, M. & Mudelsee, M. REDFIT: estimating red-noise spectra directly from unevenly spaced paleoclimatic time series. *Comput. Geosci.* **28**, 421–426 (2002).

8. Schulz, M. & Stattegger, K. Spectrum: spectral analysis of unevenly spaced paleoclimatic time series. *Comput. Geosci.* **23**, 929–945 (1997).

9. Hasselmann, K. Stochastic climate models. Part I: Theory. *Tellus* **28**, 473–485 (1976).

10. Mallat, S. A theory for multiresolution signal decomposition: the waveletrepresentation. *Pami* **11**, 674–693 (1989).

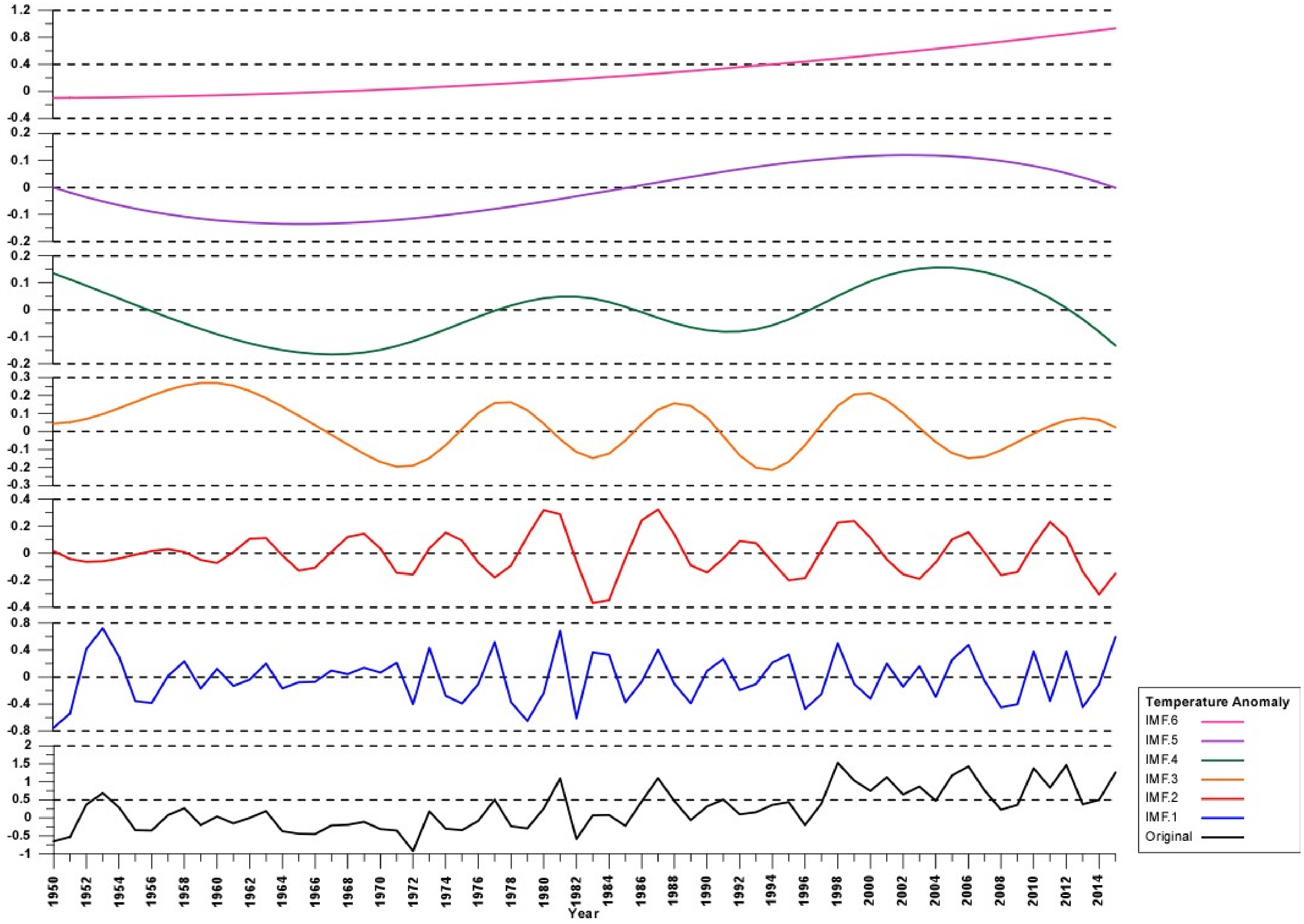

Figure S1A. The North America land temperature anomalies (TA) Intrinsic Mode Functions" (IMF) diagrams. The original signal (black line) and IMF from 1 to 6 (colored lines) was reported.

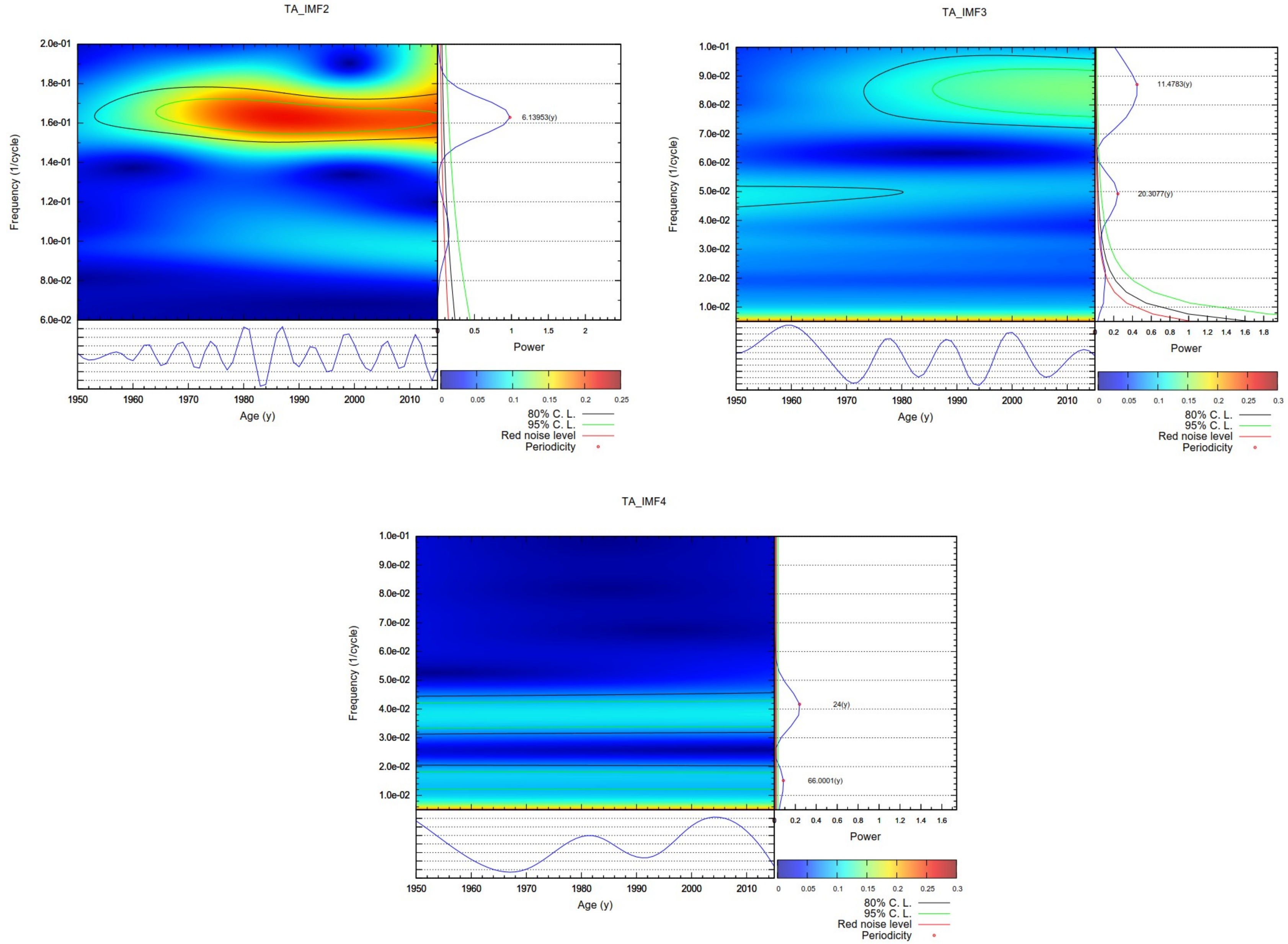

Figure S1B. The North America land temperature anomalies (TA) IMF2, 3, and 4 (horizontal boxes, blue line), Lomb-Scargle periodogram (vertical boxes, blue line), and weighted wavelet Z-transform power spectrum were reported. The green and black line represent the 95% and 80% Confident Level respectively. Significantly periodicity (red dot) and relative values expressed in years were reported.

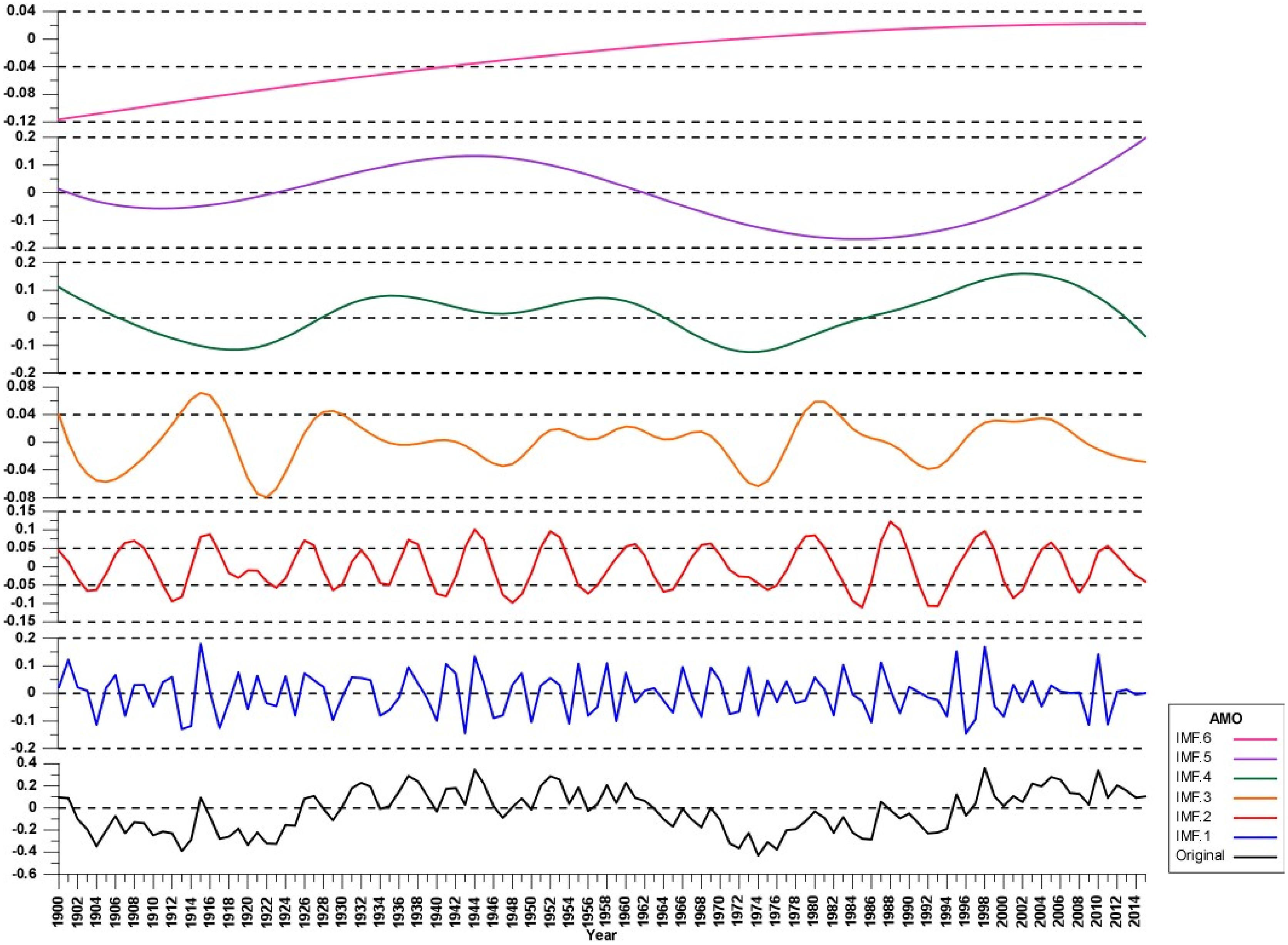

Figure S2A. The Atlantic Multidecadal Oscillation index (AMO) Intrinsic Mode Functions" (IMF) diagrams. The original signal (black line) and IMF from 1 to 6 (colored lines) was reported.

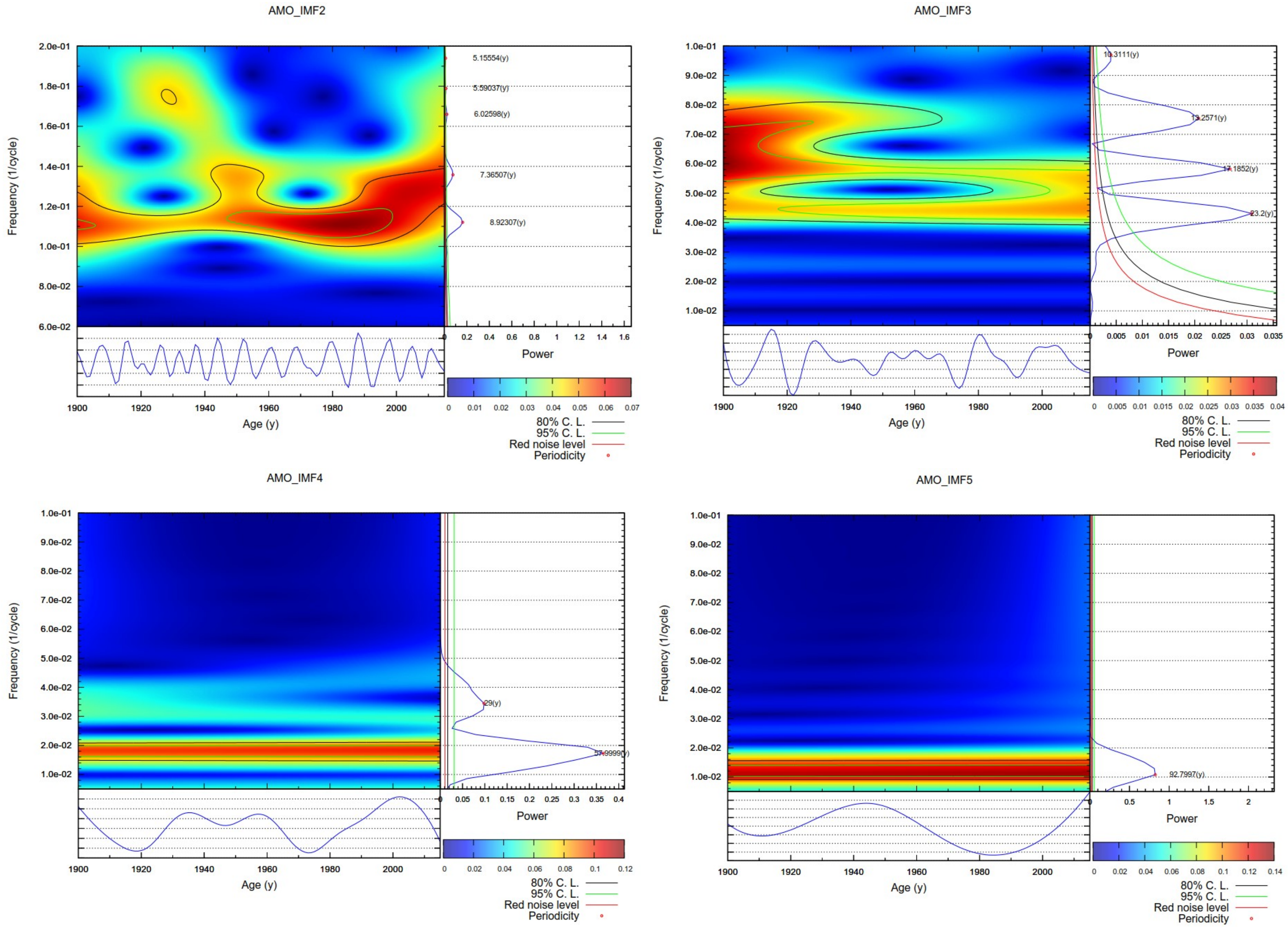

Figure S2B. The Atlantic Multidecadal Oscillation index (AMO) IMF2, 3, 4, and 5 (horizontal boxes, blue line), Lomb-Scargle periodogram (vertical boxes, blue line), and weighted wavelet Z-transform power spectrum were reported. The green and black line represent the 95% and 80% Confident Level respectively. Significantly periodicity (red dot) and relative values expressed in years were reported.

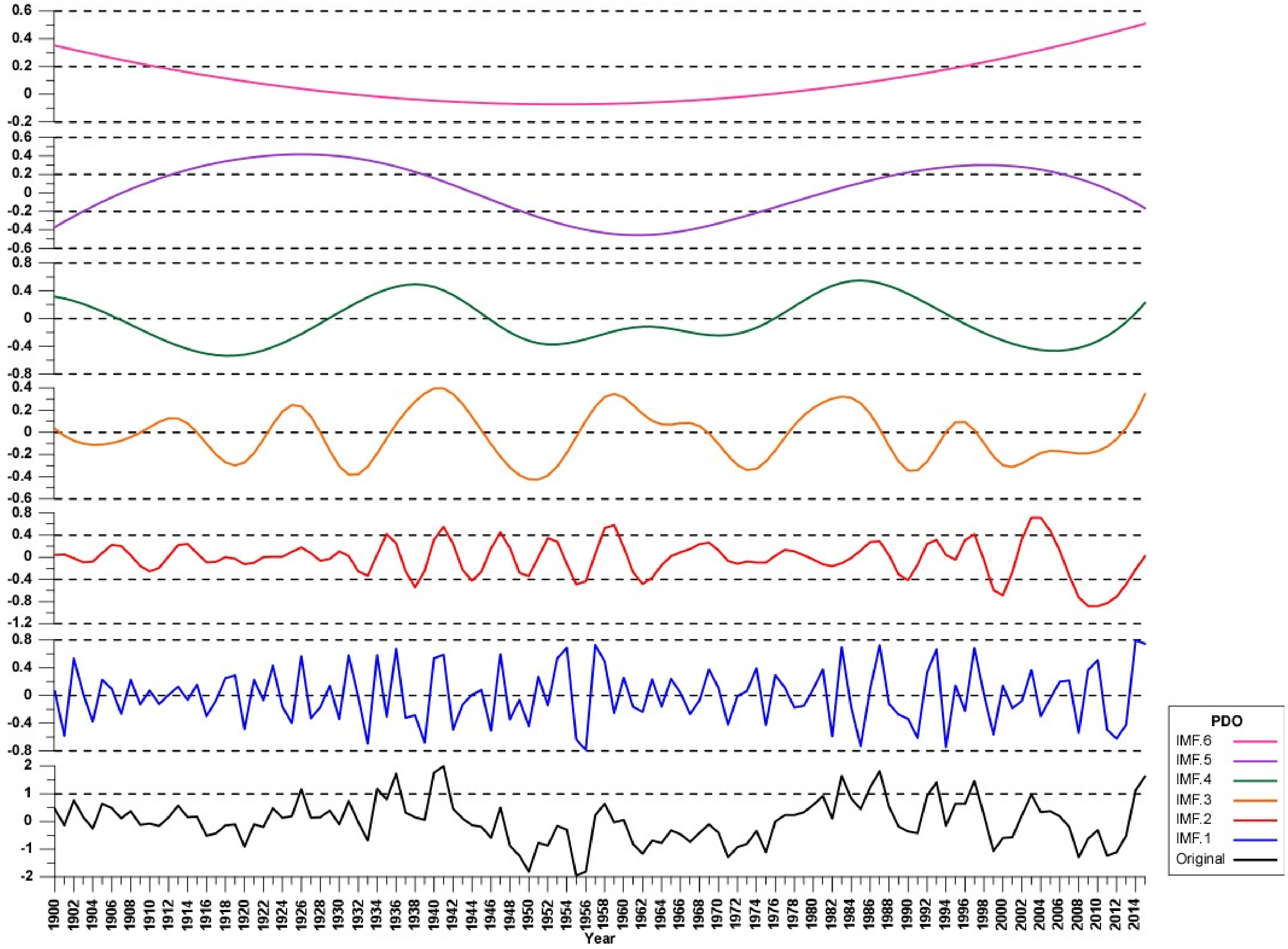

Figure S3A. The Pacific Decadal Oscillation index (PDO) Intrinsic Mode Functions” (IMF) diagrams. The original signal (black line) and IMF from 1 to 6 (colored lines) was reported.

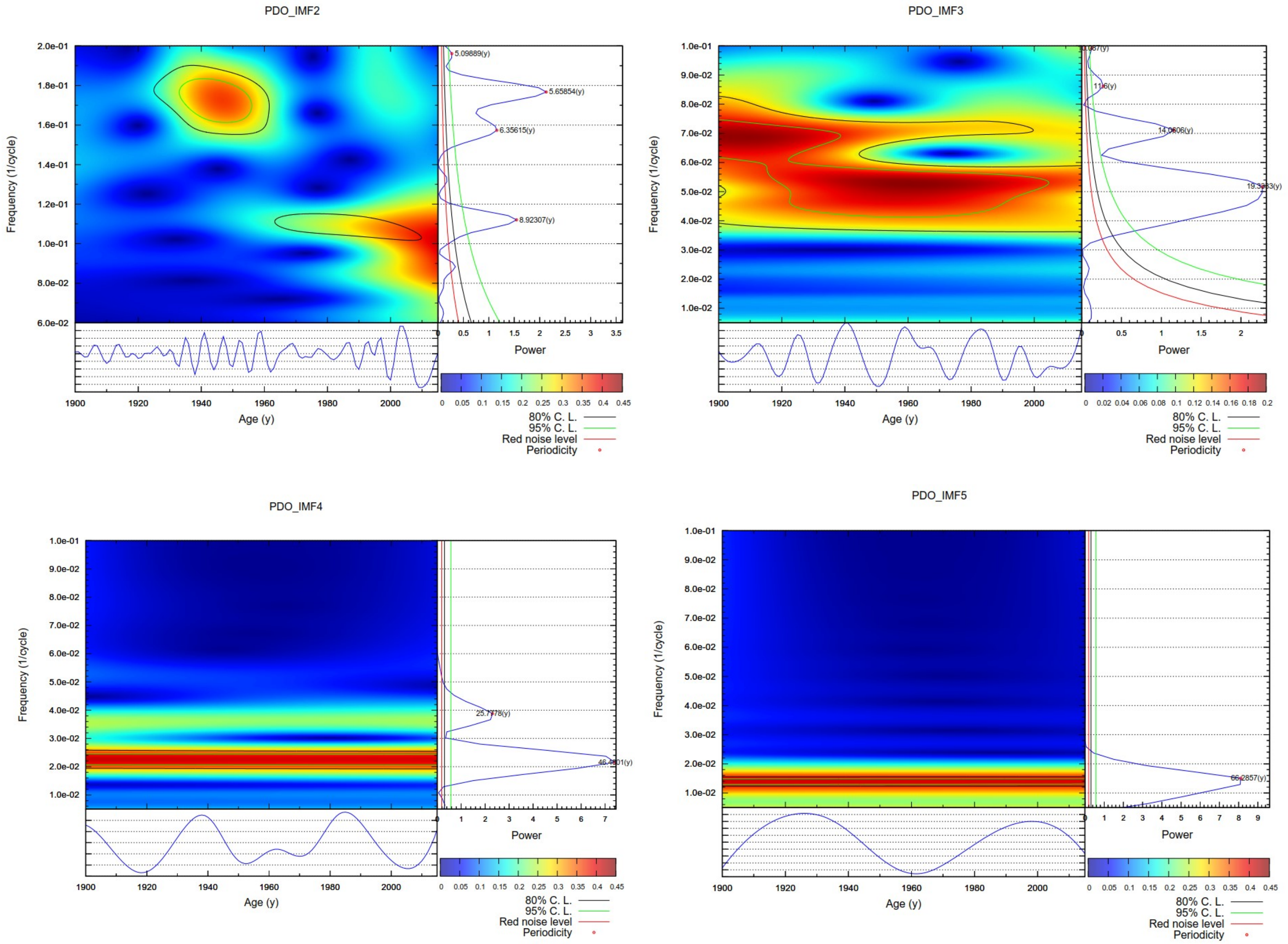

Figure S3B. The Pacific Decadal Oscillation index (PDO) IMF2, 3, 4, and 5 (horizontal boxes, blue line), Lomb-Scargle periodogram (vertical boxes, blue line), and weighted wavelet Z-transform power spectrum were reported. The green and black line represent the 95% and 80% Confident Level respectively. Significantly periodicity (red dot) and relative values expressed in years were reported.

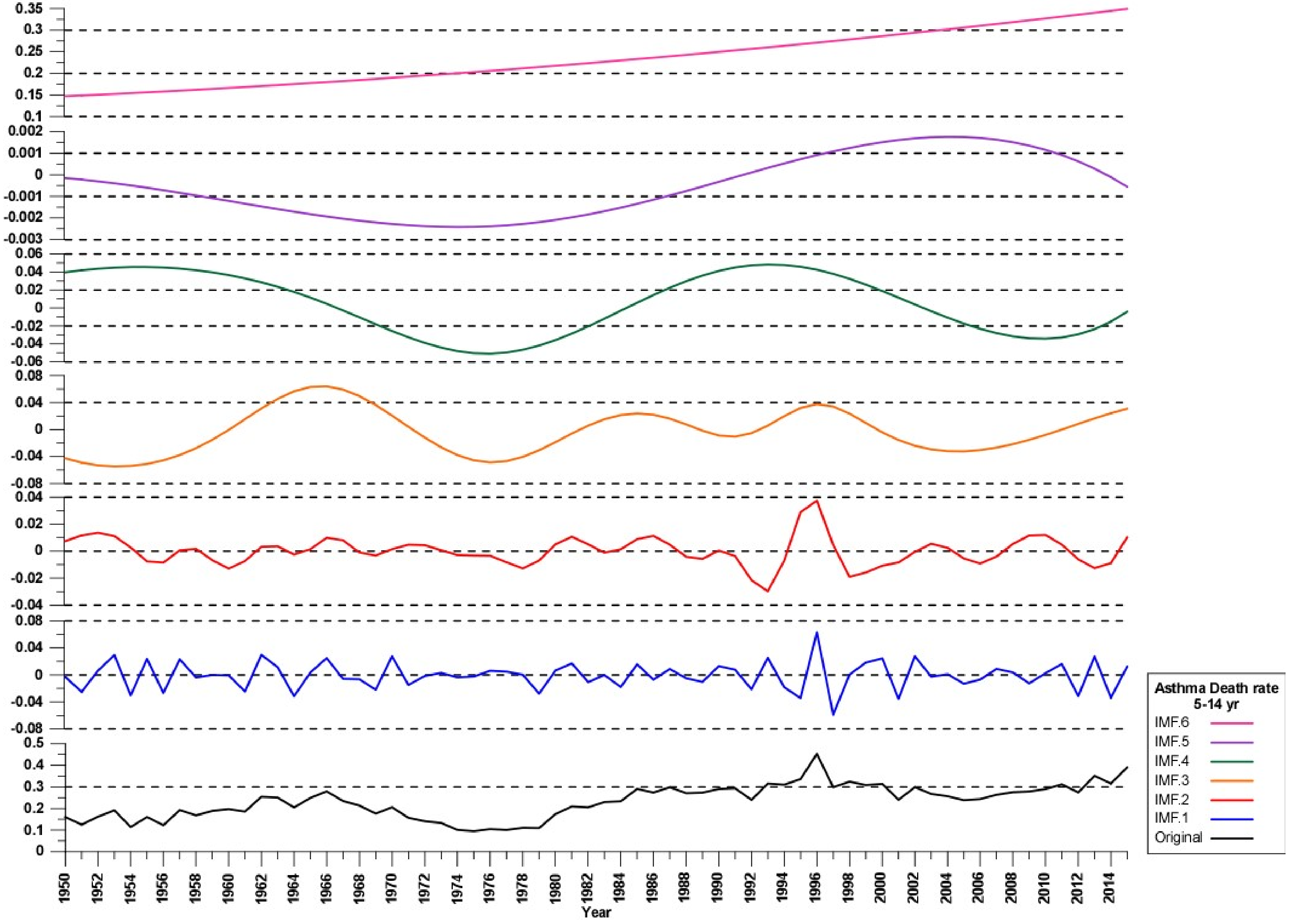

Figure S4A. Asthma death rate 5-14 yr age group Intrinsic Mode Functions" (IMF) diagrams. The original signal (black line) and IMF from 1 to 6 (colored lines) was reported.

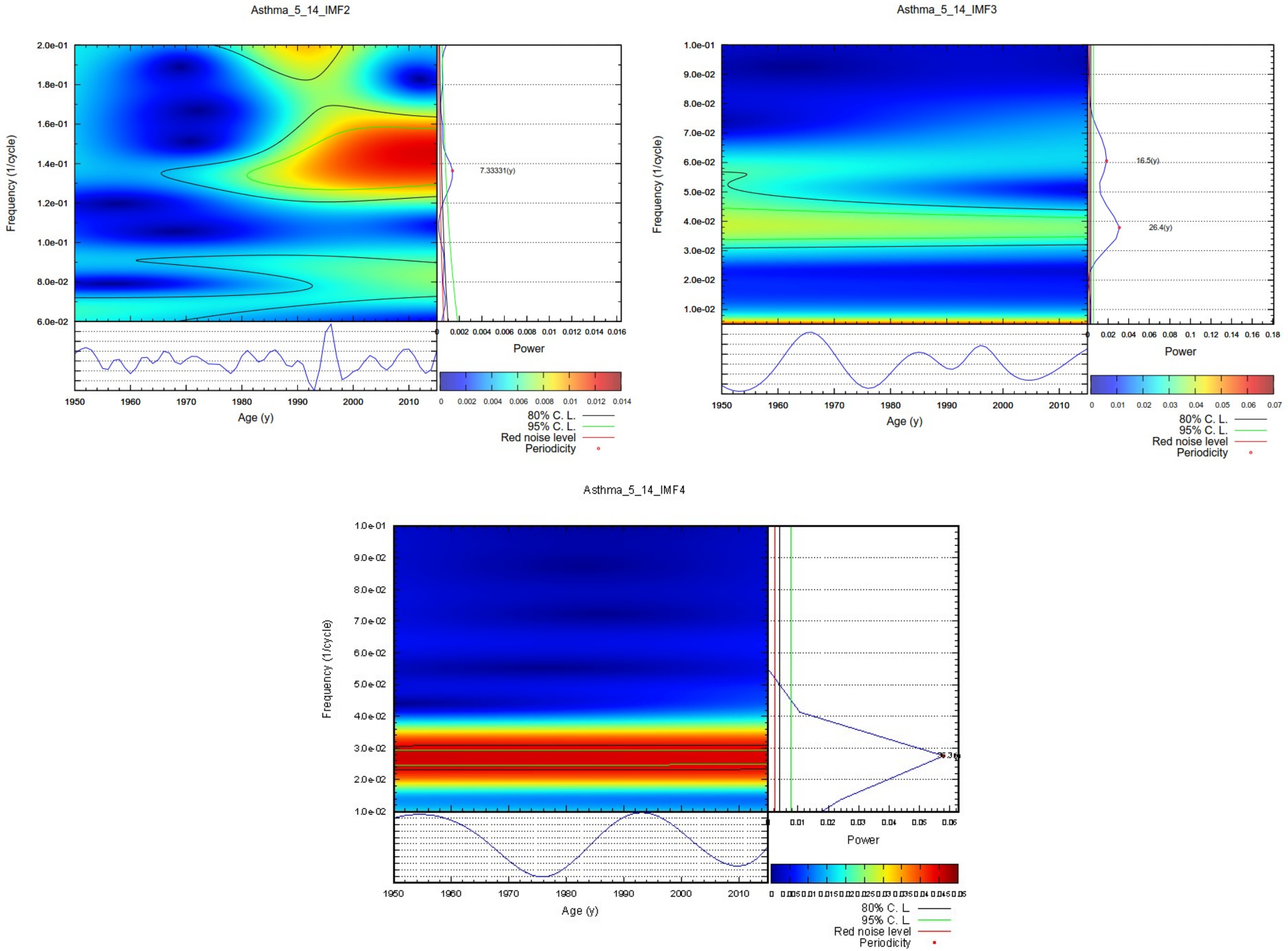

Figure S4B. Asthma death rate 5-14 yr age group IMF2, 3, and 4 (horizontal boxes, blue line), Lomb-Scargle periodogram (vertical boxes, blue line), and weighted wavelet Z-transform power spectrum were reported. The green and black line represent the 95% and 80% Confident Level respectively. Significantly periodicity (red dot) and relative values expressed in years were reported.

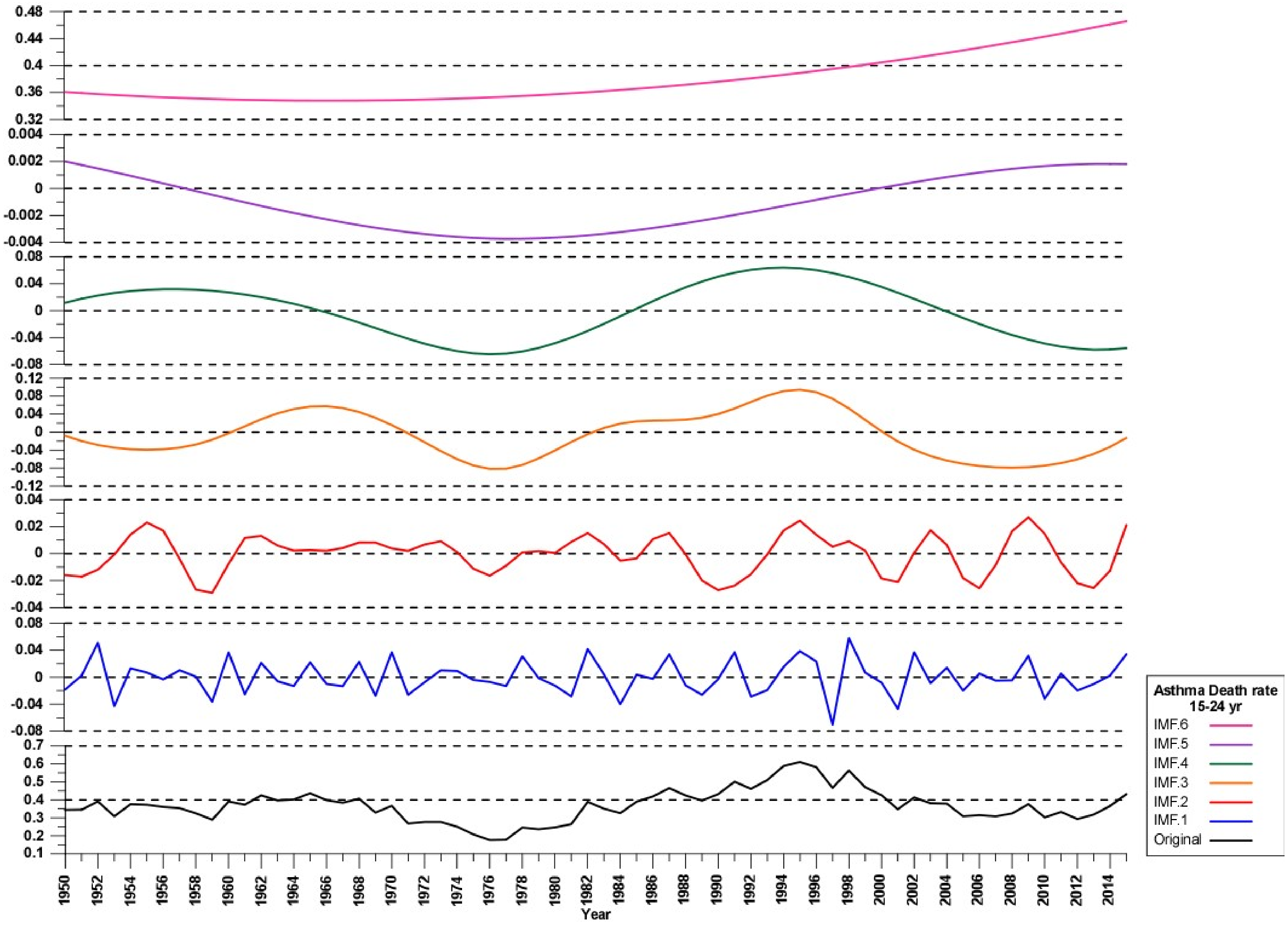

Figure S5A. Asthma mortality rate 15-24 yr age group Intrinsic Mode Functions" (IMF) diagrams. The original signal (black line) and IMF from 1 to 6 (colored lines) was reported.

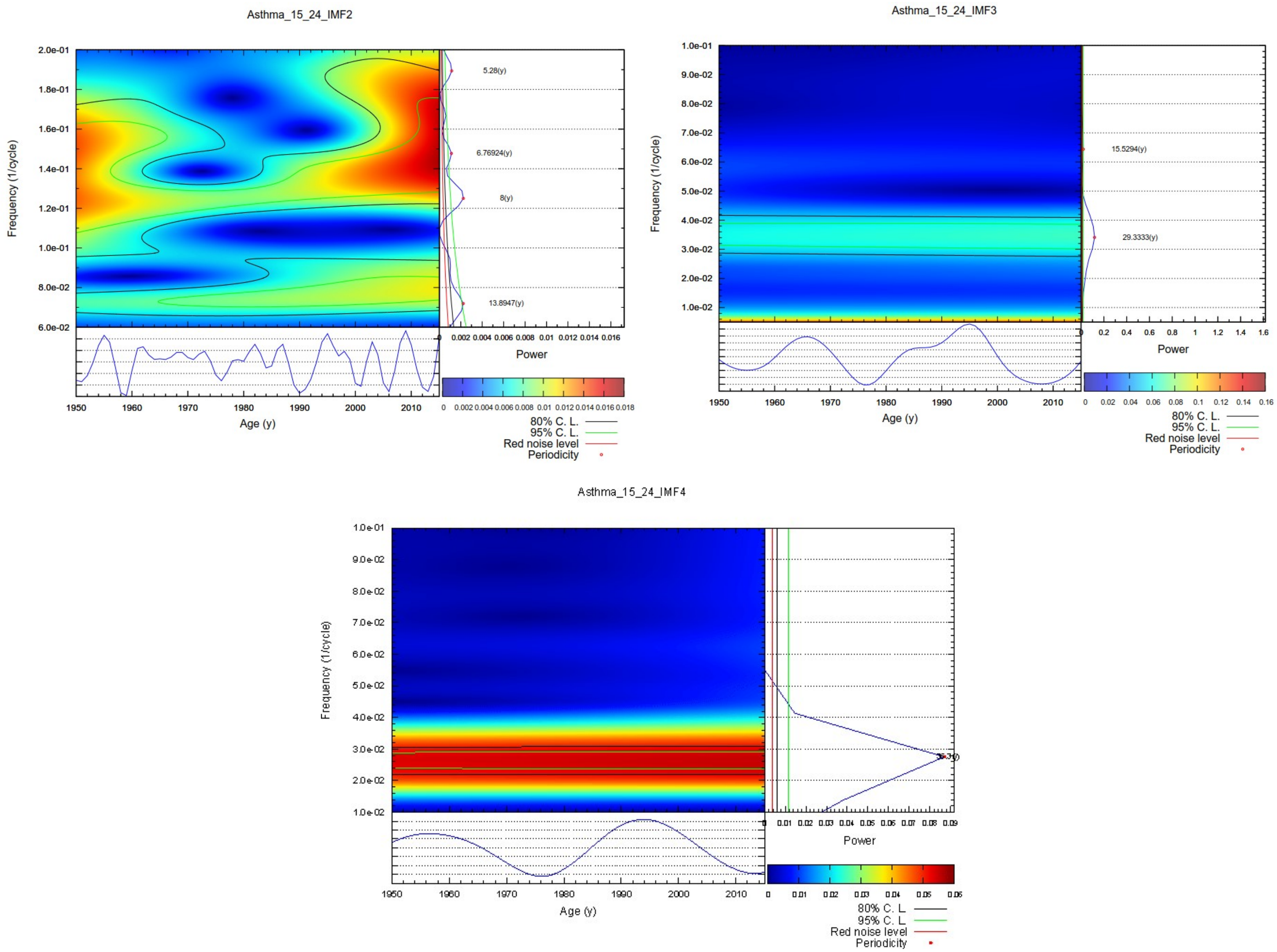

Figure S5B. Asthma mortality rate 15-24 yr age group IMF2, 3, and 4 (horizontal boxes, blue line), Lomb-Scargle periodogram (vertical boxes, blue line), and weighted wavelet Z-transform power spectrum were reported. The green and black line represent the 95% and 80% Confident Level respectively. Significantly periodicity (red dot) and relative values expressed in years were reported.

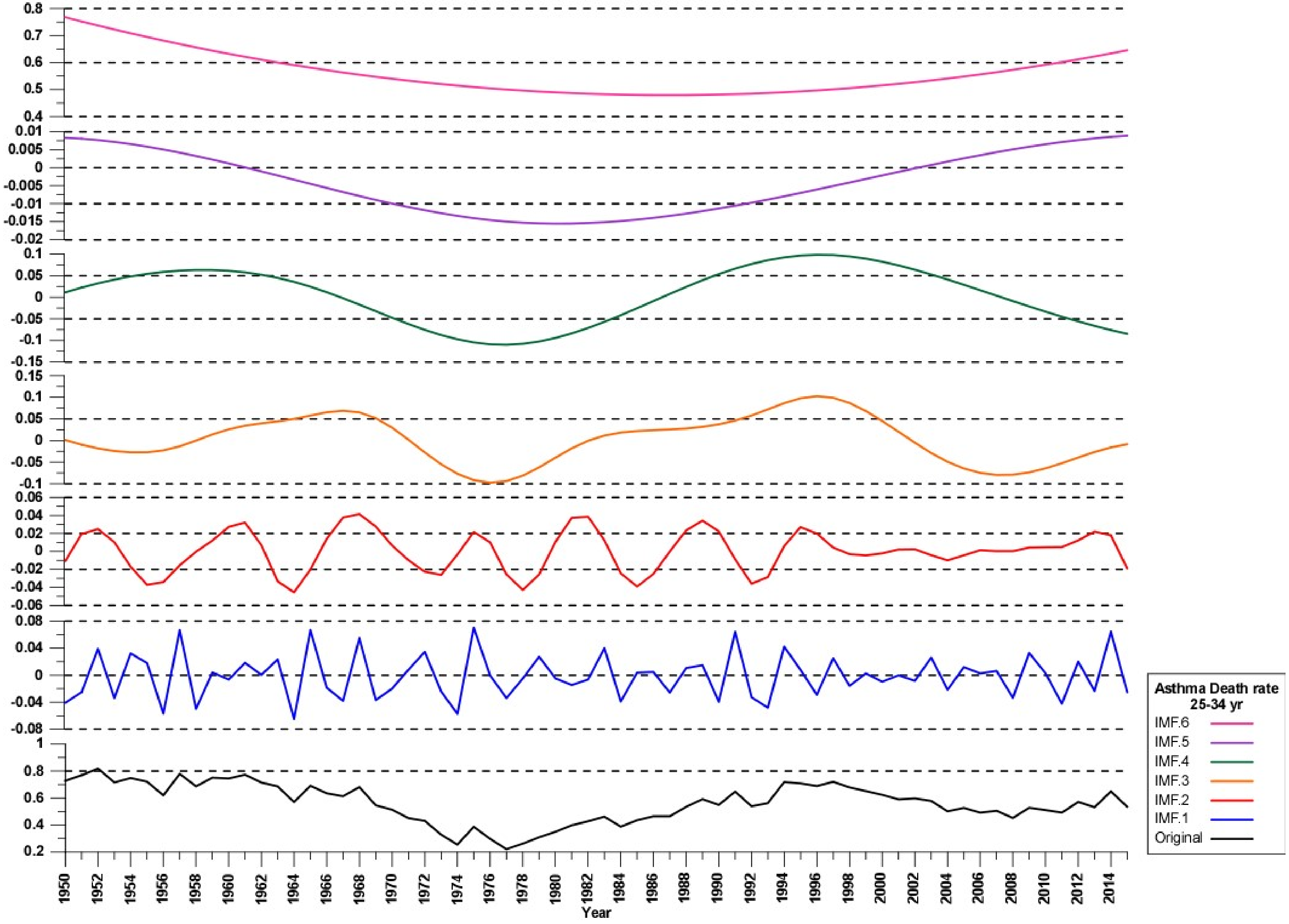

Figure S6A. Asthma mortality rate 25-34 yr age group Intrinsic Mode Functions" (IMF) diagrams. The original signal (black line) and IMF from 1 to 6 (colored lines) was reported.

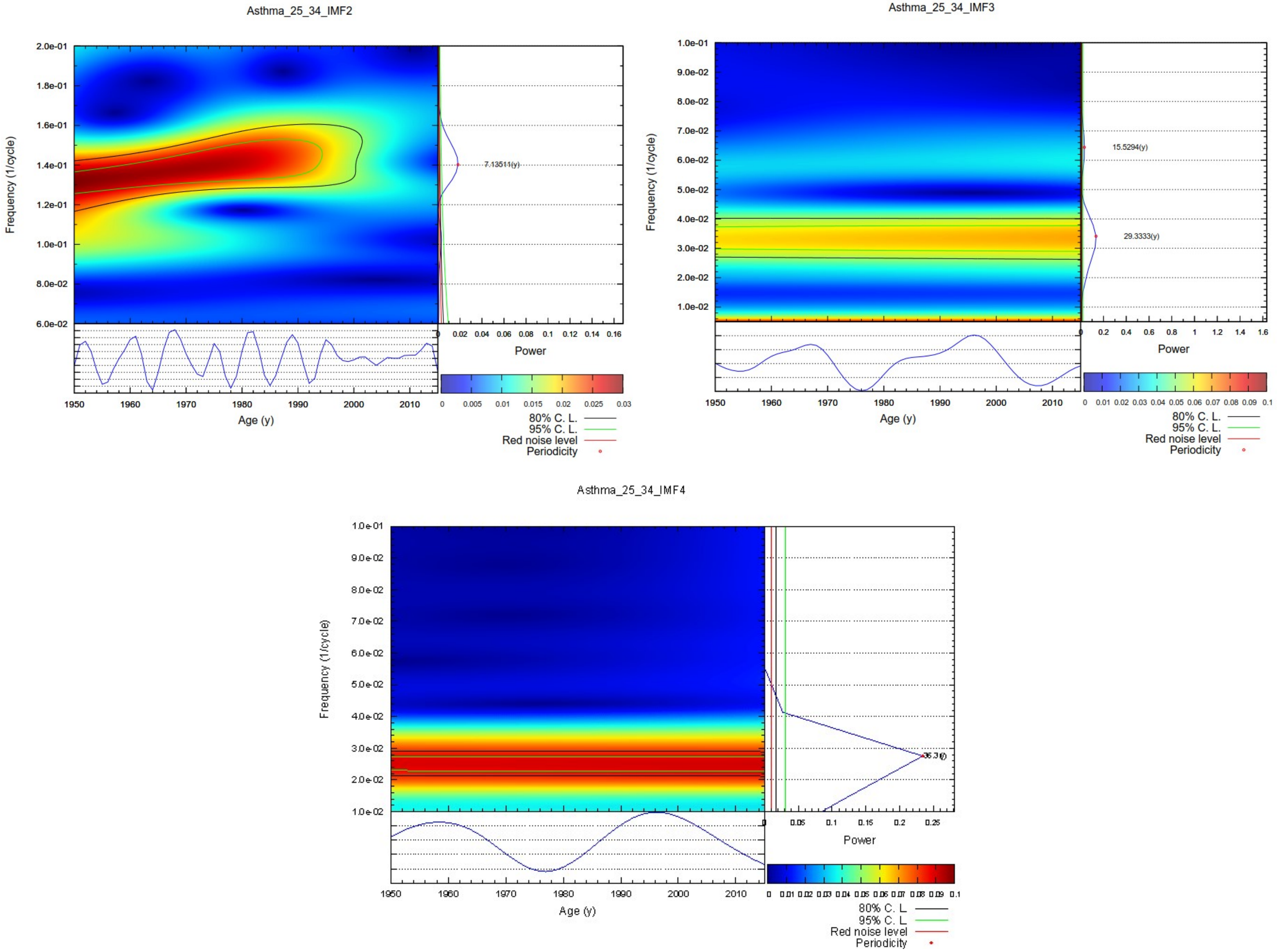

Figure S6B. Asthma mortality rate 25-34 yr age group IMF2, 3, and 4 (horizontal boxes, blue line), Lomb-Scargle periodogram (vertical boxes, blue line), and weighted wavelet Z-transform power spectrum were reported. The green and black line represent the 95% and 80% Confident Level respectively. Significantly periodicity (red dot) and relative values expressed in years were reported.

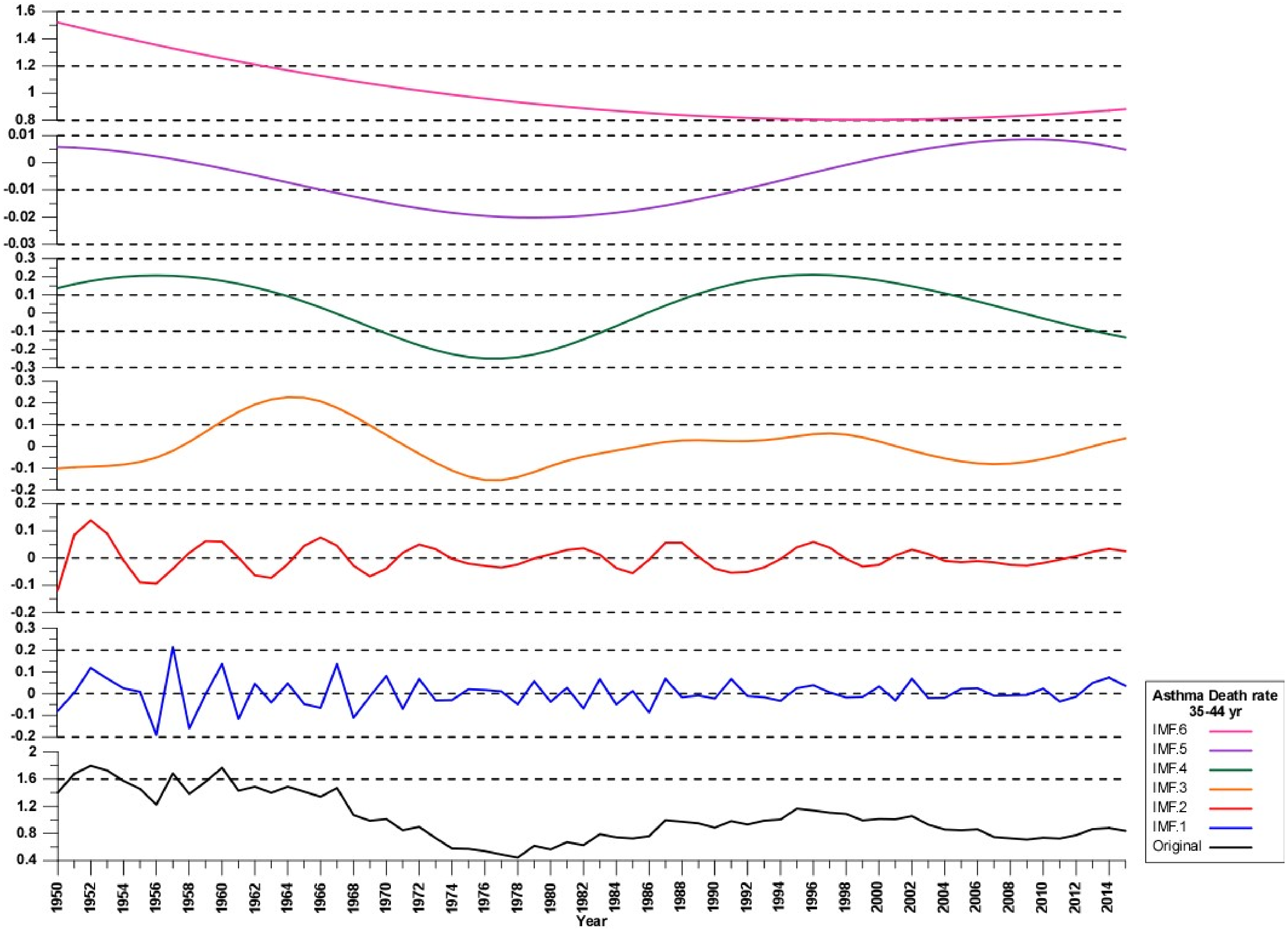

Figure S7A. Asthma mortality rate 35-44 yr age group Intrinsic Mode Functions" (IMF) diagrams. The original signal (black line) and IMF from 1 to 6 (colored lines) was reported.

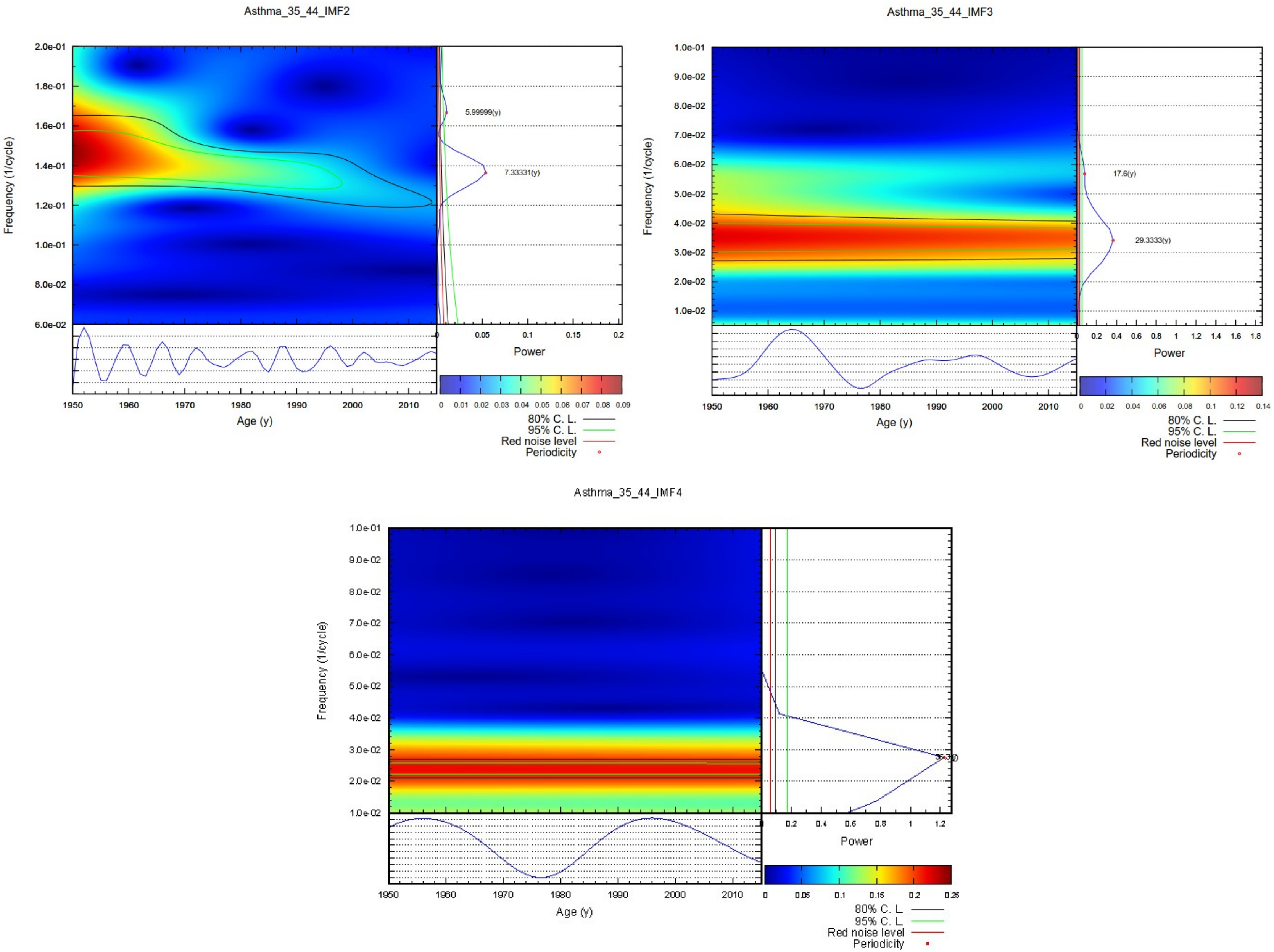

Figure S7B. Asthma mortality rate 35-44 yr age group IMF2, 3, and 4 (horizontal boxes, blue line), Lomb-Scargle periodogram (vertical boxes, blue line), and weighted wavelet Z-transform power spectrum were reported. The green and black line represent the 95% and 80% Confident Level respectively. Significantly periodicity (red dot) and relative values expressed in years were reported.
